# Characterization and Diversity of Microcystins Produced by Cyanobacteria from the Curonian Lagoon (SE Baltic Sea)

**DOI:** 10.3390/toxins13120838

**Published:** 2021-11-24

**Authors:** Donata Overlingė, Anna Toruńska-Sitarz, Marija Kataržytė, Renata Pilkaitytė, Greta Gyraitė, Hanna Mazur-Marzec

**Affiliations:** 1 Marine Research Institute, Klaipeda University, University Avenue 17, 92295 Klaipeda, Lithuania; marija.katarzyte@jmtc.ku.lt (M.K.); renata.pilkaityte@ku.lt (R.P.); greta.gyraite@apc.ku.lt (G.G.); 2 Division of Marine Biotechnology, Faculty of Oceanography and Geography, University of Gdańsk, Marszałka J. Piłsudskiego 46, PL-81378 Gdynia, Poland; anna.torunska@ug.edu.pl (A.T.-S.); hanna.mazur-marzec@ug.edu.pl (H.M.-M.)

**Keywords:** microcystin, cyanotoxin, mass spectrometry, structure elucidation

## Abstract

Microcystins (MCs) are the most widely distributed and structurally diverse cyanotoxins that can have significant health impacts on living organisms, including humans. The identification of MC variants and their quantification is very important for toxicological assessment. Within this study, we explored the diversity of MCs and their potential producers from the Curonian Lagoon. MC profiles were analyzed by liquid chromatography-tandem mass spectrometry (LC-MS/MS) method, while the potential producers were detected based on the presence of genus-specific *mcyE* gene sequences. Among the numerous MCs detected, one new potential MC variant with *m/z* 1057 was partially characterized. Moreover, two other MCs with *m/z* 1075 and *m/z* 1068 might belong to new variants with serine (Ser), rarely detected in position one of the peptides. They might also represent MC-Y(OMe)R and MC-WR, respectively. However, the application of a low-resolution MS/MS system made the unambiguous identification of the MCs impossible. Based on this example, the problems of peptide structure identification are discussed in the work. Genetic analysis revealed that potential MCs producers include *Dolichospermum*/*Anabaena*, *Microcystis* spp., and *Planktothrix agardhii*. The diversity and temporal variations in MC profiles may indicate the presence of several chemotypes of cyanobacteria in the Curonian Lagoon.

## 1. Introduction

Cyanobacteria are widely distributed oxygenic phototrophs that play an important role in the aquatic environment. Under favorable conditions, they form blooms, which have a negative effect on the ecosystem [1]. Although cyanobacteria are not considered infectious microorganisms, the toxins they synthesize have significant health impacts on living organisms, including humans [2].

The species producing cyanotoxins belong mainly to the orders *Oscillatoriales*, *Nostocales*, *Chroococcales*, and *Synechococcales* [3,4]. Among cyanotoxins, microcystins (MCs) are the most frequently studied and structurally diverse compounds [2,4,5]. To date, more than 280 variants of MCs are listed [6]. These toxins cause abnormal hepatocyte functioning leading to liver damage [7,8]. Moreover, there is growing evidence that MCs may also have reproductive and neurological effects [9,10]. Health problems occur after the ingestion of contaminated water or food, or after the absorbance of cyanotoxins through the skin during recreational activities (e.g., water sport) [7,11]. The World Health Organization (WHO) updated guideline values for MCs in drinking water (specifically for MC-LR variant—1 µg L^−1^) and water for recreational use (guideline value for MC-LR or total MCs—24 µg L^−1^) [12]. These values include the sum of all MCs in a sample [7]. However, only a limited number of MC variants as quantitative standards are available [5,13], which makes the determination of the precise value of the total MC concentration difficult. Qualitative analysis of MCs in field samples can also provide valuable data and important benefits for toxicological studies and risk assessment.

MCs are cyclic heptapeptides with the general structure of cyclo-(D-Ala^1^-X^2^-D-Masp^3^-Z^4^-Adda^5^-D-γ-Glu^6^-Mdha^7^), where Adda is 3*S*-amino-9*S*-methoxy-2*S*,6,8*S*-trimethyl-10-phenyldeca-4*E*,6*E*-dienoic acid, X and Z are variable L-amino acids, D-Masp^3^ is D-*erythro*-β-methyl-isoaspartic acid, and Mdha is *N*-methyldehydroalanine [14,15]. The structural modifications can occur in all seven amino acids, but they are mainly recorded in positions 2 and 4 [5,7]. The one-letter amino acid code of two residues is included in the symbols of a specific MC variant (e.g., MC-LR has leucine (L) and arginine (R) in positions 2 and 4, respectively) [5]. MCs are synthesized by the nonribosomal peptide synthetase and polyketide synthase encoded by the *mcyA-J* genes. The organization of the *mcy* cluster was resolved in the representants of the main MCs producing genera: *Dolichospermum* [16], *Microcystis* [17], and *Planktothrix* [18]. *Mcy* genes are frequently used as genetic markers for the detection, differentiation, and identification of MC producers [19,20]. *McyE* is responsible for Adda synthesis, as well as for the addition of D-glutamate into the MC molecule [19]. As this part of the MC structure is crucial for the activity of the compound, *mcyE* has been most widely used to assess the risk associated with cyanobacteria blooms [21]. Apart from general primers used for the amplification of the *mcyE* gene, the presence of toxic *Dolichospermum*, *Microcystis*, and *Planktothrix* genera in the environment has been studied with genus-specific *mcyE* primers [19,22].

Data on the presence of MC-producing cyanobacteria, MCs diversity (including other cyanometabolites), and their concentrations have also been reported from the Curonian Lagoon located in the southeastern part of the Baltic Sea [23,24,25,26,27]. The lagoon is a hyper-eutrophic, mainly freshwater body, connected to the Baltic Sea by the narrow Klaipėda Strait. In the lagoon, summer cyanobacteria blooms are an annual phenomenon that has been reported for several decades [28]. Most of the cyanobacteria species found in the lagoon belong to fresh–brackish water (42%) and freshwater species (37%) [29]. *Aphanizomenon* spp. is a dominant species in the Curonian Lagoon, co-occurring with *Planktothrix agardhii*, *Microcystis* spp., *Dolichospermum*/*Anabaena* spp., and *Woronichinia* spp. [23,24,28]. Dense cyanobacteria blooms are more frequently observed in the southern and central parts than in the northern part of the water body [28]. MC concentrations in the coastal southern part occasionally reach the guideline values recommended by the WHO, posing a higher risk to humans [24]. In the Russian part of the Curonian Lagoon, the MCs producing cyanobacteria have been detected using molecular methods [30,31]. In both studies, *mcyE* genes and cyanotoxins were associated with the presence of *Microcystis* species.

The aim of this work was to determine and document the diversity of MCs produced by cyanobacteria from the Curonian Lagoon, and identify the source organisms using genus-specific primers. In order to detect and characterize the structure of 20 MC variants present in the field samples, liquid chromatography-tandem mass spectrometry (LC-MS/MS) was applied. Genetic analysis showed that at least part of the cyanobacteria belonging to the *Dolichospermum*/*Anabaena*, *Microcystis*, and *Planktothrix* genera are MC producers.

## 2. Results

### 2.1. Cyanobacteria Community

**Figure 1:**
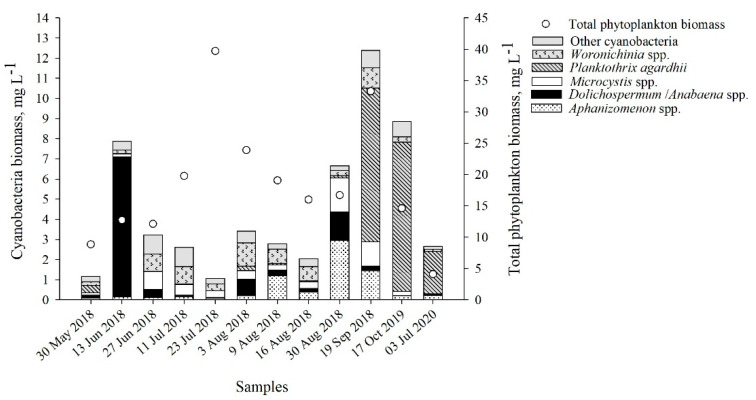
Structure and biomass (mg L^−1^) of the cyanobacteria community and total phytoplankton biomass (mg L^−1^) in the collected samples.

In samples collected from the shore station of the Curonian Lagoon, cyanobacteria biomass varied from 1.17 to 12.39 mg L^−1^, and accounted for 3–65% of the total phytoplankton biomass (TPB) (Figure 1, Table S2). During most of the summer (27 June 2018–16 August 2018), the composition of cyanobacteria species did not differ (Figure S1), while late spring–early summer and early autumn were characterized by different communities of cyanobacteria. *Dolichospermum flosaquae* dominated in June (71% of the total cyanobacteria biomass (TCB)), while the contribution of *Planktothrix agardhii* was highest in July (2020), September–October (62–84% of the TCB).

**Figure 2:**
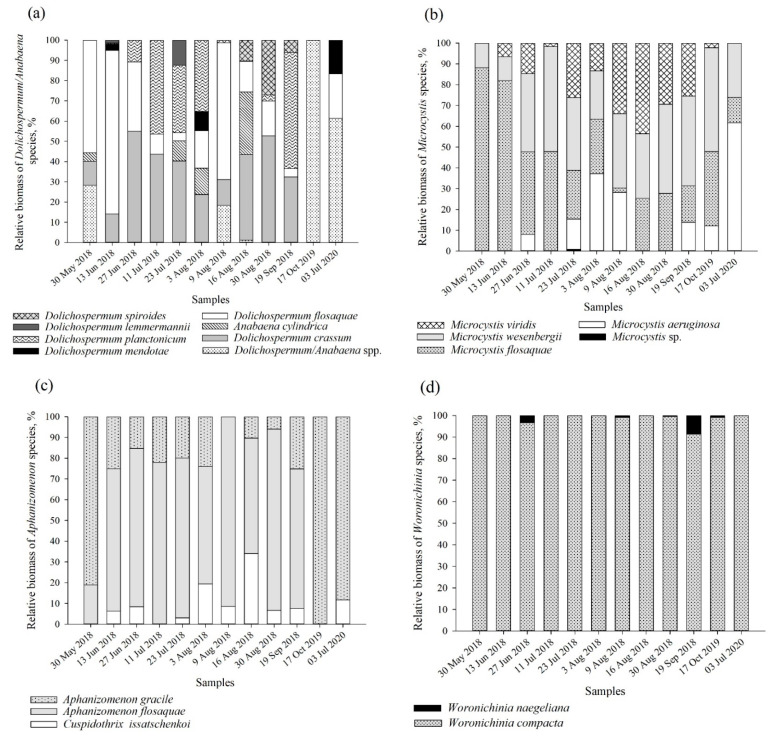
Relative biomass of dominating cyanobacteria species of the genera *Dolichospermum*/*Anabaena* (**a**), *Microcystis* (**b**), *Aphanizomenon* (**c**) and *Woronichinia* (**d**). Relative biomass is calculated from the total biomass of specific genera.

The greatest species diversity (eight species) and the differences in relative biomass were found among members of the genus *Dolichospermum* (Figure 2a). Together with *D. flosaquae*, the dominance of *D. crassum* and *D. planctonicum* was recorded (<57% of the TB of the genus *Dolichospermum*) (Figure 2a, Table S2). No evident seasonal differences in the occurrence of *M. flosaquae* and *M. wesenbergii* were observed. The two species were found in all samples, and their contribution to the total biomass was similar in most of the samples (2–88% and 23–51% of the TB of the genus *Microcystis*, respectively) (Figure 2b). A more pronounced dominance of *M. flosaquae* was observed during late spring–early summer (30 May–13 June) (82–88% of the TB of the genus *Microcystis*). *M. viridis* and *M. aeruginosa* represented a slightly different seasonal pattern: the highest contribution of the species biomass was recorded during the middle of the summer (July–August) (26–43% and 28–61% of the TB of the genus *Microcystis*). *Aphanizomenon* genus was mainly represented by *Aph. flosaquae* (0–91% of the TB of the genus *Aphanizomenon*), together with *Aph. gracile* and *Cuspidothrix issatschenkoi* (Figure 2c). *Woronichinia compacta* was the dominant species among representatives of the *Woronichinia* genus (91–100% of the TB of the genus *Woronichinia*) and was present in all samples (Figure 2d).

### 2.2. Microcystins Diversity

In total, 20 MC variants were detected in the samples, most commonly with 2–5 different variants per sample (Table 1, Figures S3–S22). Of these, MC-RR was detected in all samples. Other frequently detected MCs were MC-LR and [Dha^7^]MC-RR, which were present in 11 and 7 out of 12 samples, respectively. The following variants belonged to the most rarely recorded: ([Ser^1^]MC-HtyR (or MC-Y(OMe)R), MC-WR (or [Ser^1^]MC-HarR), MC-(H4)TyrR, MC-HphR, [Asp^3^]MC-RY, MC-FR, MC-LW, MC-HilR, MC-LY, and [Asp^3^]MC-LY; they were found in only one or two samples. The abbreviations of the amino acids and their full names are provided in Table S1. The highest number of MC variants was detected during July 2018 (12 variants) and in the second half of August 2018 (18 variants). 

**Figure 3:**
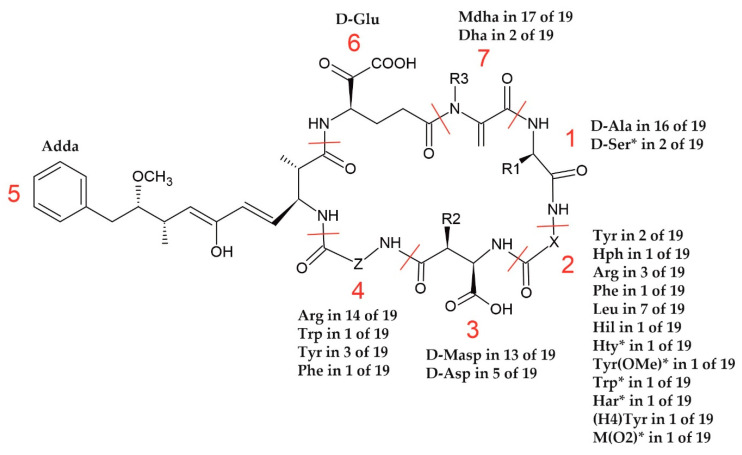
A general structure of MCs and their structural diversity recorded in the samples from the Curonian Lagoon (R1 = CH_3_ or CH_2_OH; R2 = H or CH_3_; R3 = H or CH_3_; X and Z—variable L-amino acids). * One of the possible residues. See the text in Section 2.2. The abbreviations of the amino acids and their full names are provided in Table S1.

In position 1 of the MCs produced by cyanobacteria from the Curonian Lagoon, D-Ala was mainly present (in 16 of the 19 MCs) (Figure 3). There were also two MC structures, with *m/z* 1075 and *m/z* 1068, where the presence of Ser was suspected. The amino acid residues in position 2 were the most diverse amongst the MCs detected in the Curonian Lagoon samples. In position 2, Leu was found most frequently (7/19), followed by Arg (3/19). The other amino acid residues were present in two (Tyr) or one (Hph, Phe, Hil, Hty, Tyr(OMe), Trp, Har, (H_4_)Tyr, M(O2)) MC variant. Position 4 was more conserved and was occupied mainly by Arg (14/19) or optionally by Tyr (3/19), Phe (1/19), and Trp (1/19). In positions 3 and 7, methylated or dimethylated analogues of D-Asp and Dha were present, respectively. No modifications were observed in positions 5 and 6.

**Figure 4:**
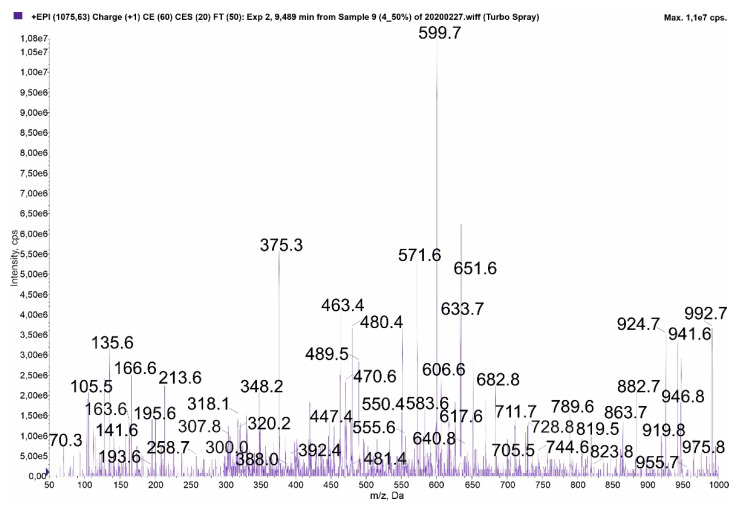
Enhanced product ion mass spectrum of MC with *m/z* 1075. The spectrum can correspond to [Ser^1^]MC-HtyR (I) or MC-Y(OMe)R (II). The structure elucidation of [Ser^1^]MC-HtyR (I) was based on the following fragment ions: *m/z* 992 [M + H – Mdha]; 946 [M + H – Glu/Masp]; 941 [M + H – Adda fragment]; 924 [C_11_H_14_O + Glu + Mdha + Ser + Hty + Masp + Arg + H]; 919 [M + H – Arg]; 882 [Masp + Arg + Adda + Glu + Mdha + H]; 863 [Ser + Hty + Masp + Arg + Adda + H]; 728 [Masp + Arg + Adda + Glu + H]; 682 [Arg + Adda + Glu + Mdha + H]; 633 [Mdha + Ser + Hty + Masp + Arg + H]; 606 [Glu + Mdha + Ser + Hty + Masp + H]; 599 [Arg + Adda + Glu + H], 571 [Arg + Adda + Glu + H – CO], 550 [Ser + Hty + Masp + Arg + H]; 470 [Arg + Adda + H]; 463 [C_11_H_14_O + Glu + Mdha + Ser + H]/[Hty + Masp + Arg + H]; 375 [C_11_H_14_O + Glu + Mdha + H]; 348 [C_11_H_14_O + Glu + Mdha + H – CO]/[Mdha + Ser + Hty + H]; 307 [Hty + Masp + H]; 300 [Glu + Mdha + Ser + H]; 213 [Glu + Mdha + H]; 163 [C_11_H_14_O + H]; 135 Adda fragment.

The collected spectra of three MCs contain fragment ions that can be generated by two different variants: [Ser^1^]MC-HtyR/MC-Y(OMe)R (*m/z* 1075) (Figure 4), MC-WR/[Ser^1^]MC-HarR (*m/z* 1068) (Figure S4), and [Asp^3^]MC-YR/[Asp^3^]MC-M(O_2_)R (*m/z* 1031) (Figure S12). These peptides differ only in positions 1 and 2. Since the total value of the two residues present in different variants is the same (e.g., Ser^1^+Hty^2^ and Ala^1^+Tyr(OMe)^2^; Ala^1^+Trp^2^ and Ser^1^+Har^2^; Ala^1^+Tyr^2^ and Ala^1^-M(O2)^2^), the fragment ions containing both residues are also the same. The encountered problems are illustrated in Figure 4, Figures S4 and S12.

MC-Y(OMe)R (II) structure elucidation was based on the following fragment ions: *m/z* 992 [M + H – Mdha]; 946 [M + H – Glu/Masp]; 941 [M + H – Adda fragment]; 924 [C_11_H_14_O + Glu + Mdha + Ala + Tyr(OMe) + Masp + Arg + H]; 919 [M + H – Arg]; 882 [Masp + Arg + Adda + Glu + Mdha + H]; 863 [Ala + Tyr(OMe) + Masp + Arg + Adda + H]; 728 [Masp + Arg + Adda + Glu + H]; 682 [Arg + Adda + Glu + Mdha + H]; 633 [Mdha + Ala + Tyr(OMe) + Masp + Arg + H]; 606 [Glu + Mdha + Ala + Tyr(OMe) + Masp + H]; 599 [Arg + Adda + Glu + H], 571 [Arg + Adda + Glu + H – CO], 550 [Ala + Tyr(OMe) + Masp + Arg + H]; 480 [Tyr(OMe) + Masp + Arg + H]; 470 [Arg + Adda + H]; 447 [C_11_H_14_O + Glu + Mdha + Ala + H]; 375 [C_11_H_14_O + Glu + Mdha + H]; 348 [C_11_H_14_O + Glu + Mdha + H – CO]/[Mdha + Ala + Tyr(OMe) + H]; 213 [Glu + Mdha + H]; 163 [C_11_H_14_O + H]; 135 Adda fragment.

### 2.3. Genetic Analysis

To identify the possible producers of MCs in the Curonian Lagoon, the polymerase chain reactions (PCRs) were performed with *mcyE* genus-specific primers in addition to *mcyE* general primers. When general primers were used, the PCR products were obtained for all the environmental samples and the two MCs producing cyanobacteria strains *M. aeruginosa* CCNP1102 and *P. aghardii* CCNP1325 (Table 2). The application of *Microcystis*- and *Planktothrix*-specific primers also gave products in all the bloom samples and the relevant cyanobacteria strains. Only for the sample collected on 17 October 2019, no amplification product with *Dolichospermum*-specific *mcyE* primers was detected.

Sequences of the received PCR products were deposited in GenBank under the accession numbers OK500398-OK500432. Sequences obtained in amplification using *Microcystis*-specific primers were 100% similar and grouped with other sequences from potentially toxic *M. flos-aquae*, *M. viridis* of different origin, and one sequence from *Pseudanabaena* sp. CCM-UFV065 (Figure S2). Sequences received from PCRs with *Planktothrix*-specific primers grouped with sequences obtained from *P. aghardii*, *P. rubescens*, and one sequence from *Synechococcus* sp., while those obtained from *Dolichospermum*-specific grouped with other *Dolichospermum*/*Anabaena* sequences.

## 3. Discussion

In our studies, *Aph. flosaquae*, *M. wesenbergii*, and *W. compacta* form a significant part of the total cyanobacteria biomass [23,32]. The *Aph. flosaquae* population from the Curonian Lagoon, similarly to the one from the Baltic Sea, is considered non-MC producing [33]. During its blooms, no significant increases in MC concentrations and diversity were observed [23,27]. In the recent work by Österholm et al. [34], *mcy* genes were not detected in several analyzed *Aph. flosaquae* genomes. *M. wesenbergii* is rarely considered as an MC producer, and to date, only one work showing *mcyE* gene amplification from this species has been published [35]. As for the *Woronichinia* genus, the presence of MCs in bloom samples dominated by some species, e.g., *W. naegeliana*, has been occasionally recorded [36,37], but no data on toxin production by isolated *W. compacta* strain were published. Based on the reviewed data, we can conclude that although the biomass of *Aph. flosaquae*, *M. wesenbergii*, and *W. compacta* were high, these species do not contribute to the toxicity of blooms in the Curonian Lagoon.

Phytoplankton analyses of samples collected in the Curonian Lagoon showed a frequent occurrence of *Dolichospermum*/*Anabaena*, *Microcystis*, and *Planktothrix* genera among the potential producers of MCs. This was also reflected by the presence of *mcyE* genes amplified with primers specific for these genera. Furthermore, *P. agardhii* is the only species among the *Planktothrix* genus found in the Curonian Lagoon [23], and according to our genetic analyses, at least part of the population belongs to MC producers. *Microcystis*, *Dolichospermum*/*Anabaena*, and *Planktothrix* are frequently found in temperate zones, and usually, during their presence, different variants of MCs are detected [4,35,38,39,40,41]. However, assigning a particular MC variant to any of the cyanobacteria genera or species is impossible, especially because the study was performed with the application of field samples with a mixture of cyanobacteria species. This becomes even more complicated by the fact that one cyanobacteria strain can produce a median number of 4–5 MCs simultaneously, with one or two variants being dominant in any single strain [42,43]. However, based on the most identified associations between cyanobacteria and MCs [18,44,45,46], *M. flosaquae*, *D. flosaquae*, and *P. agardhii*, which were found in almost all samples, potentially can be considered as the main producers of the most frequently detected MC-RR and MC-LR and their demethylated variants ([Asp^3^]MC-RY, [Dha^7^]MC-RR, [Asp^3^]MC-LY, [Asp^3^]MC-LR).

Sensitive molecular methods, based on *mcy* genes, were developed to detect and identify MC producers [19,47,48]. Unfortunately, as a result of the observed *mcy* genes deletions, recombinations, and various insertions, the presence of the *mcy* genes cannot be directly linked to the synthesis of MCs. Furthermore, the influence of biotic and abiotic factors on *mcy* gene expression is still poorly recognized, and the knowledge about the changes in the process in given genotypes is limited [19,49]. Studies show that among tested *Dolichospermum* spp. (=*Anabaena*) (126 strains from the Baltic Sea), *Planktothrix* (72 strains from European lakes), and *Microcystis* spp. (18 strains from worldwide), only in 1–13% of the isolates *mcy* genes were not expressed [47,48,50]. Due to a relatively low percentage of *mcy* genes carrying but not expressing genera, the screening of *mcy* genes for the identification of potential MC producers from field samples may give a good qualitative assessment. However, as a single parameter, these genes cannot provide a clear conclusion; therefore, the application of other methods, such as mass spectrometry, must be considered for the detection of MCs.

During our study, 20 different MC variants were characterized based on fragmentation spectra. Among the MCs with a fully elucidated structure, in only two variants the presence of Ser in position 1 was considered, while in others D-Ala was present. This result is in line with previous findings that indicated position 1 as highly conserved and predominantly occupied by Ala. According to Bouaïcha et al. [51], D-Ala^1^ was present in 219 of the 279 identified MC variants, while only two D-Ser^1^-containing variants were reported. The diversity and frequency of other amino acids in specific positions of MCs from the Curonian Lagoon, as in the case of position 1, correspond to the previous structural studies of the toxins [4,51,52]. Positions 2 and 4 were most variable, while modifications in other positions were minor.

In some cases, structure elucidation based on the product ion fragmentation spectrum was found to be difficult, and could lead to misinterpretation of the spectrum. This problem can be encountered when residues with the same value are present in the peptide, e.g., Glu and Masp, Mdha and Dhb, Leu and Ile, or Tyr and Met(O_2_). MCs with Met(O_2_) can be formed during sample processing, and are considered post-extraction oxidation artifacts [53]. This fact cannot be excluded in our work, either. In addition, the sequence of two or more residues can produce the products with the same *m/z* value in the spectrum. The structure identification in this study was additionally hampered by the low resolution of the QTRAP5500 system (and *m/z* range limited to 1000). In general, structure elucidation of peptides based on fragmentation pattern, even if the mass spectrum is rich in fragment ions, might be tentative and always should be performed with caution. Moreover, in the case of new variants, the structure elucidation should be confirmed by nuclear magnetic resonance spectroscopy (NMR). However, if sufficient amounts of pure MC variants for NMR analysis cannot be isolated, the application of high-resolution mass spectrometry and accurate mass measurement can provide new data for more reliable structure elucidation.

The highest diversity of MCs was observed in samples collected on 23 July 2018 (12 variants) and 16 August 2018 (18 variants), when the contribution of cyanobacteria to TPB was one of the lowest. Compared to other studies, the diversity of MCs detected during our study is relatively higH—Yilamaz et al. [54] reported more than 36 MCs detected in Turkish lake, Ballot et al. [55] detected 41 MC variants in a dam located in South Africa, and Fastner et al. [46] reported 15 MC variants in German freshwaters. The species composition in the samples collected on 23 July 2018 and 16 August 2018 did not differ evidently from other samples collected during the period from 27 June to 30 August. The significant differences in MC profiles recorded in these samples indicate changes at the sub-population level of the cyanobacteria community. In water bodies, several cyanobacteria chemotypes characterized by different MC patterns usually coexist [56,57,58]. In the Curonian Lagoon, the environmental conditions on the two days when the highest MC structural diversity was recorded (23 July and 16 August) apparently favored the proliferation of MC-rich chemotypes, and thus, indirectly influenced the presence of numerous, including the more rare, MC variants (e.g., MC-HtyR, [Ser^1^]MC-RR, MC-LW, or MC-LY). Environmental conditions have an impact on the structure and dynamics of the cyanobacteria community, but not necessarily on the production of different MC variants [59,60]. Moreover, diversity can increase as a result of relaxation of the adenylation domain [50]. Recombination patterns in the adenylation domains might lead to the synthesis of new MCs [61,62]. More detailed research with isolated strains is needed to clarify the diversity of cyanobacteria chemotypes in the Curonian Lagoon.

The samples from the Curonian Lagoon also displayed diversity and considerable variation in rare and potentially new MC variants throughout the sampling period. MC variants, such as MC-WR, [Asp^3^]MC-RY, MC-LW, MC-LY, MC-(H_4_)YR, and [Asp^3^]MC-LY, detected during our study are mainly produced by representatives of the *Microcystis* genus [63,64,65,66,67]. Furthermore, MC-FR and MC-HilR were detected in the blooms dominated by several *Microcystis* species [68,69], while MC-HphR is associated with different strains of *Anabaena* [53,70]. The list of MCs detected in cyanobacteria bloom samples from the Curonian Lagoon and presented in this study adds new information that can be useful in establishing the geography of specific MC variants. Geographical differences in the distribution of specific MC variants are observed worldwide. For example, Leu^1^-containing MCs were detected only in Canada [71], while MC-LA is more frequently detected in the US rather than in European water bodies [72].

The identification of MC variants and their accurate quantification are very important for toxicological assessment and monitoring [73]. The following MCs: MC-LR, MC-HilR, MC-LY, MC-YR, belong to the highly toxic variants [51]. For other MCs, including the new variants, no toxicity data are available [51]. Moreover, due to the lack of reference standards, the reliable quantitative assessment of total MCs is impossible. It should be also noted that MCs are not the only harmful substances produced by cyanobacteria. There are many other classes of peptides (anabaenopeptins, cyanopeptolins, aeruginosins, etc.), which occur as frequently as MCs, and are mainly known as proteases inhibitors or compounds harmful against grazers [5]. Lack of toxicological data and comprehensive knowledge about the activity of cyanobacteria metabolites might lead to the underestimation of the real risk.

## 4. Conclusions

This study showed for the first time the diversity of MCs produced by cyanobacteria from the Curonian Lagoons. While genetic analyses indicated the potential of the cyanobacteria to produce MCs, the chemical analyses allowed us to obtain more conclusive results. The detection of 20 different MCs (among which three might be potentially new variants) adds new information about their geographical distribution and may indicate the presence of different cyanobacteria chemotypes in the lagoon.

## 5. Materials and Methods

### 5.1. Water Samples

Phytoplankton samples were collected from the shore of the Curonian Lagoon, located in the city of Nida (sampling site coordinates 55.300115, 22.005821) during 2018 (10 samples, every two weeks from May to September), 2019 (1 sample, October), and 2020 (1 sample, July). For DNA analysis, water samples were collected in 2018 from the surface layers (~50 cm) in sterile, darkened plastic bottles. The samples were filtered (0.5–1.0 L; the sample volume depended on the abundance of cyanobacteria) through a 0.22 µm pore size mixed cellulose ester filters (MontaMil^®^ Membrane Filters, Frisenette ApS, Knebel, Denmark) for genetic analysis and GF/F glass fiber filters (Whatman International Ltd., Kent, UK) for MC analysis. Visually, the biomass collected on filters was similar for all samples (intensely green color).

### 5.2. Phytoplankton Analysis

Phytoplankton samples for microscopic analysis were taken during all sampling events and fixed with acid Lugol’s iodine solution. Quantitative analyses of the composition of the phytoplankton community were conducted using a LEICA DMI 3000 inverted microscope (Leica Microsystems CMS, Wetzlar, Germany) at magnifications of ×100 and ×400, according to the method described by Utermöhl [74]. Phytoplankton was identified to the lowest possible taxonomic level using guidelines described in the literature for the freshwater environments [75,76,77,78]. According to HELCOM recommendations [79], the phytoplankton abundance (counts L^−1^) was calculated by multiplying the number of units counted (filamentous cyanobacteria were counted in lengths of 100 μm as one count) with the coefficient C (L), calculated using the following equation:

(1) $$C\;=\frac{A\;\times 1000}{N\;\times a\;\times V}$$

where A is the cross-section area of the top cylinder of the combined sedimentation chamber (the usual inner diameter is 25.0 mm, giving A = 491 mm^2^), N is the number of counted fields or transects, a is the area of a single field or transect, and V is the volume (mL) of sedimented aliquot.

The biomass of phytoplankton (mg L^−1^) was calculated by the allocation of phytoplankton species (genus) to size classes, according to the scheme of Olenina et al. (2006) and updated appendix available at HELCOM website (https://helcom.fi/helcom-at-work/projects/peg/, accessed on 5 September 2021). The biomass of phytoplankton (mg L^−1^) was calculated based on the following equation, as recommended by HELCOM [79]:

Biomass = abundance × VCU × 10^−6^, (2)

where VCU is the volume of the counting unit (µg).

### 5.3. DNR Extraction and PCR

DNA extraction was performed using the PowerWater^®^ DNA Isolation Kit (MO BIO Laboratories, Inc., Carlsbad, CA, USA) and FastDNA^TM^ Spin Kit for Soil (MP Biomedicals, Santa Ana, CA, USA) (samples collected during 2018); DNeasy PowerSoil Pro Kit (QIAGEN, Hilden, Germany) (samples collected during 2019 and 2020). The quality and quantity of extracted DNA were determined with SpectraMax^®^ i3 Platform (Molecular Devices LLC., Sunnyvale, CA, USA) equipped with SpectraDrop Micro-Volume Microplate. PCR products were detected by electrophoresis in 1.5% agarose gel stained with SYBR^®^ Green I (Sigma-Aldrich, St. Louis, MO, USA). DNA isolated from toxic *M. aeruginosa* CCNP1102 and *P. aghardii* CCNP1305 were used as a positive control [57,80]. DNA isolated from *Limnoraphis* sp. CCNP1324, and MilliQ water were used as a negative control [81].

For amplification of the *mcyE* gene from all cyanobacteria present in the samples, the same primers (*mcyE-F2* and *mcyE-R4*) (Genomed S.A., Warszawa, Poland) and PCR cycling conditions were used as in Rantala et al. [82]. MC-producing *Anabaena/Dolichospermum*, *Microcystis*, and *Planktothrix* spp. were targeted with the above-mentioned forward primer and genus-specific reverse primers (*mcyE-12R*, *mcyE-R8*, and *mcyE-plaR3*, respectively) (Genomed S.A., Warszawa, Poland) [19,83]. PCRs were run in 25 μL solution containing approx. 100 ng of DNA, 5 pmol of each specific oligonucleotide primer, 12.5 μL of MyTaq™ Red Mix (Bioline Reagents Ltd., London, UK), in Mastercycler^®^ nexus GSX1 (Eppendorf, Hamburg, Germany).

The PCR products were purified with an ExtractMe DNA clean-up kit (Blirt S.A., Gdańsk, Poland). The PCR fragments were sequenced (Genomed S.A., Warszawa, Poland) using both forward and reverse genus-specific primers used in the amplification. The obtained nucleotide sequences were edited with Chromas Lite 2.1, aligned and assembled, and afterward compared to the sequences in the NCBI GenBank (http://www.ncbi.nlm.nih.gov) using blast algorithm (http://blast.ncbi.nlm.nih.gov). They were deposited under OK500398–OK500432 accession numbers. For phylogenetic analyses, the sequences were aligned using the MEGA version X [84], the alignments were corrected manually. Neighbor-joining (NJ) and maximum likelihood (ML) trees were constructed in MEGA version X. For each tree, a bootstrap analysis of 1000 replications was performed.

### 5.4. Microcystins Analysis

The collected material was extracted with 75% methanol. Filters were sonicated with an ultrasonic disrupter (1 min) (HD 2070 Sonopuls, Bandelin, Berlin, Germany), then in the water bath (15 min) (Sonorex, Bandelin, Berlin, Germany), and centrifuged (12,000 *g*; 15 min) (Eppendorf 5810R, Hamburg, Germany). The extracts were analyzed using an Agilent HPLC system (Agilent Technologies, Waldboronn, Germany) coupled to a hybrid triple quadrupole/linear ion trap mass spectrometer QTRAP LC-MS/MS (QTRAP5500, Applied Biosystems, Sciex; Toronto, ON, Canada), according to the method described by Mazur-Marzec et al. [85]. The presence of MCs was screened using information dependent acquisition (limit of detection was 0.5–2.0 ng mL^−1^, depending on the variant). Data were processed with Analyst QS (Version 1.5.1, Applied Biosystems/MDS Analytical Technologies, Concord, ON, Canada, 2008).

### 5.5. Statistical Analysis

Non-parametric multidimensional scaling (nMDS) based on the Bray–Curtis similarity coefficient [86] was used to represent the similarities of dominating cyanobacteria communities among the different samples. The stress values of the two MDS plots were determined—a stress value of <0.2 indicated an accurate representation of similarity rankings. nMDS was conducted using Primer v6 software (PRIMER-E Ltd., Plymouth, UK).

## Figures and Tables

**Table 1:** MC diversity in field samples collected from the Curonian Lagoon during 2018, 2019, and 2020 (“+”: detected; empty cells: not detected, *m/z*—values of MC pseudomolecular ions. In brackets, the value of a doubly charged ion is given).

MC Variants	*m/z*	Sampling Dates											
		30 May 2018	13 Jun 2018	27 Jun 2018	11 Jul 2018	23 Jul 2018	3 Aug 2018	9 Aug 2018	16 Aug 2018	30 Aug 2018	19 Sep 2018	17 Oct 2019	3 Jul 2020
[Ser^1^]MC-HtyR or MC-Y(OMe)R	1075					+			+				
MC-WR or [Ser^1^]MC-HarR	1068								+	+			
MC-X^1^R	1057					+			+				
MC-?	1054								+				
MC-(H_4_)YR	1049					+			+				
MC-YR	1045	+				+			+	+	+		
MC-HphR	1043								+	+			
MC-RR	1038 (519)	+	+	+	+	+	+	+	+	+	+	+	+
[Asp^3^]MC-YR or [Asp^3^]MC-M(O_2_)R	1031					+		+	+	+			
[Asp^3^]MC-RY	1031											+	+
MC-FR	1029					+			+				
MC-LW	1025								+				
[Dha^7^]MC-RR	1024 (512)		+			+			+	+	+	+	+
MC-HilR	1009					+			+				
MC-LY	1002								+				
MC-LR	995	+	+	+	+	+	+		+	+	+	+	+
[Asp^3^]MC-LY	988								+			+	
MC-LF	986						+		+	+	+		
[Dha^7^]MC-LR	981		+			+			+	+			
[Asp^3^]MC-LR	981					+						+	+

Note: Unknown part of MC.

**Table 2:** The results of PCR amplification with the general *mcy* primers and *Dolichospermum*-, *Microcystis*-, and *Planktothrix*-specific primers (“+”—PCR-positive results, “−”—PCR-negative results, OK500398-OK500432—accession number in GenBank).

Samples	General *mcyE* Primers	*Dolichospermum* Specific Primers	*Microcystis* Specific Primers	*Planktothrix* Specific Primers
30 May 2018	+	OK500398	OK500409	OK500421
13 Jun 2018	+	OK500399	OK500410	OK500422
27 Jun 2018	+	OK500400	OK500411	OK500423
11 Jul 2018	+	OK500401	OK500412	OK500424
27 Jul 2018	+	OK500402	OK500413	OK500425
3 Aug 2018	+	OK500403	OK500414	OK500426
9 Aug 2018	+	OK500404	OK500415	OK500427
16 Aug 2018	+	OK500405	OK500416	OK500428
30 Aug 2018	+	OK500406	OK500417	OK500429
19 Sep 2018	+	OK500407	OK500418	OK500430
17 Oct 2019	+	−	OK500419	OK500431
3 Jul 2020	+	OK500408	OK500420	OK500432
*M. aeruginosa* CCNP1102	+	−	OK500396	−
*P. aghardii* CCNP1325	+	−	−	OK500397
*Limnoraphis* sp. CCNP1324	−	−	−	−
MilliQ water	−	−	−	−

## Data Availability

The data presented in this study are available in Supplementary Material.

## Supplementary Materials

The following are available online at https://www.mdpi.com/article/10.3390/toxins13120838/s1, Table S1: The abbreviations of amino acids and their full names, Table S2: Presence and dominance of cyanobacteria species in the samples from the Curonian Lagoon. Cyanobacteria dominance was expressed as a percentage of total cyanobacteria biomass, Figure S1: nMDS plot based on Bray–Curtis similarity matrix of dominating cyanobacteria species biomass. Only samples for which concentrations of microcystins were calculated in µg L^−1^ were used for RDA analysis, Figure S2: Phylogenetic relationships of sequences retrieved from the studied environmental samples (marked in colors), their closest relatives and representants of other potent microcystin producing genera, based on the alignment of partial (218 b) *mcyE* sequences. Numbers above branches indicate the bootstrap values for NJ and ML method. Accession numbers in NCBI are presented in brackets, Figure S3: Enhanced product ion mass spectrum of microcystin with the suggested structure [Ser^1^]MC-HtyR (*m/z* 1075) and the following fragment ions: *m/z* 992 [M + H – Mdha]; 946 [M + H – Glu/Masp]; 941 [M + H – Adda fragment]; 924 [C_11_H_14_O + Glu + Mdha + Ser + Hty + Masp + Arg + H]; 919 [M + H – Arg]; 882 [Masp + Arg + Adda + Glu + Mdha + H]; 863 [Ser + Hty + Masp + Arg + Adda + H]; 728 [Masp + Arg + Adda + Glu + H]; 682 [Arg + Adda + Glu + Mdha + H]; 633 [Mdha + Ser + Hty + Masp + Arg + H]; 606 [Glu + Mdha + Ser + Hty + Masp + H]; 599 [Arg + Adda + Glu + H], 571 [Arg + Adda + Glu + H – CO], 550 [Ser + Hty + Masp + Arg + H]; 470 [Arg + Adda + H]; 463 [C_11_H_14_O + Glu + Mdha + Ser + H]/[Hty + Masp + Arg + H]; 375 [C_11_H_14_O + Glu + Mdha + H]; 348 [C_11_H_14_O + Glu + Mdha + H – CO]/[Mdha + Ser + Hty + H]; 307 [Hty + Masp + H]; 300 [Glu + Mdha + Ser + H]; 213 [Glu + Mdha + H]; 163 [C_11_H_14_O + H]; 135 Adda fragment; or enhanced product ion mass spectrum of microcystin with the suggested structure MC-Y(OMe)R (*m/z* 1075), and the following fragment ions: *m/z* 992 [M + H – Mdha]; 946 [M + H – Glu/Masp]; 941 [M+H – Adda fragment]; 924 [C_11_H_14_O + Glu + Mdha + Ala + Tyr(OMe) + Masp + Arg + H]; 919 [M + H – Arg]; 882 [Masp + Arg + Adda + Glu + Mdha + H]; 863 [Ala + Tyr(OMe) + Masp + Arg + Adda + H]; 728 [Masp + Arg + Adda + Glu + H]; 682 [Arg + Adda + Glu + Mdha + H]; 633 [Mdha + Ala + Tyr(OMe) + Masp + Arg + H]; 606 [Glu + Mdha + Ala + Tyr(OMe) + Masp + H]; 599 [Arg + Adda + Glu + H], 571 [Arg + Adda + Glu + H – CO], 550 [Ala + Tyr(OMe) + Masp + Arg + H]; 480 [Tyr(OMe) + Masp + Arg + H]; 470 [Arg + Adda + H]; 447 [C_11_H_14_O + Glu + Mdha + Ala + H]; 375 [C_11_H_14_O + Glu + Mdha + H]; 348 [C_11_H_14_O + Glu + Mdha + H – CO]/[Mdha + Ala + Tyr(OMe) + H]; 213 [Glu + Mdha + H]; 163 [C_11_H_14_O + H]; 135 Adda fragment, Figure S4: Enhanced product ion mass spectrum of microcystin with the suggested structure MC-WR (*m/z* 1068) and the following fragment ions: *m/z* 997 [M + H – Ala]; 985 [M + H – Mdha]; 939 [M + H – Glu/Masp]; 934 [M + H – Adda fragment]; 917 [M + H – Adda fragment – H_2_O]; 912 [M + H – Adda]; 856 [M + H – (Glu + Mdha)]; 811 [M + H – (Ala + Trp)]; 728 [Masp + Arg + Adda + Glu + H]; 682 [Arg + Adda + Glu + Mdha + H]; 626 [Mdha + Ala + Trp + Masp + Arg + H]; 599 [Arg + Adda + Glu + H], 582 [Agr + Adda + Glu + H – NH_3_]; 571 [Masp + Arg + Adda + H – CO]/[Arg + Adda + Glu + H – CO]; 543 [Ala + Trp + Masp + Arg + H]; 526 [Adda + Glu + Mdha + H]; 472 [Trp + Masp + Arg + H]; 470 [Arg + Adda + H]; 456 [Trp + Masp + Arg + H – NH_3_]; 375 [C_11_H_14_O + Glu + Mdha + H]; 347 [C_11_H_14_O + Glu + Mdha + H – CO]; 341 [Mdha + Ala + Trp + H]; 269 [Masp + Arg + H – NH_3_]; 258 [Ala + Trp + H]; 213 [Glu + Mdha + H]; 195 [Glu + Mdha + H – H_2_O]; 135 Adda fragment; or enhanced product ion mass spectrum of microcystin with the suggested structure [Ser^1^]MC-HarR (*m/z* 1068), and the following fragment ions: *m/z* 997 [M + H – Ala]; 985 [M + H – Mdha]; 939 [M + H – Glu/Masp]; 934 [M + H – Adda fragment]; 917 [M + H – Adda fragment – H_2_O]; 912 [M + H – Adda]; 856 [M + H – (Glu + Mdha)]; 811 [M + H – (Ser + Har)]; 728 [Masp + Arg + Adda + Glu + H]; 682 [Arg + Adda + Glu + Mdha + H]; 626 [Mdha + Ser + Har + Masp + Arg + H]; 599 [Arg + Adda + Glu + H], 582 [Agr + Adda + Glu + H – NH_3_]; 571 [Masp + Arg + Adda + H – CO]/[Arg + Adda + Glu + H – CO]; 543 [Ser + Har + Masp + Arg + H]; 526 [Adda + Glu + Mdha + H]; 470 [Arg + Adda + H]; 456 [Har + Masp + Arg + H]; 375 [C_11_H_14_O + Glu + Mdha + H]; 347 [C_11_H_14_O + Glu + Mdha + H – CO]; 341 [Mdha + Ser + Har + H]; 300 [Har + Masp + H]/[Glu + Mdha + Ser + H]; 258 [Ser + Har + H]; 213 [Glu + Mdha + H]; 195 [Glu + Mdha + H – H_2_O]; 135 Adda fragment, Figure S5: Enhanced product ion mass spectrum of microcystin with the partially elucidated structure MC-XR (*m/z* 1057) and the following fragment ions: 728 [Masp + Arg + Adda + Glu + H]; 682 [Arg + Adda + Glu + Mdha + H]; 599 [Arg + Adda + Glu + H], 571 [Arg + Adda + Glu + H – CO], 470 [Arg + Adda + H]; 375 [C_11_H_14_O + Glu + Mdha + H]; 195 [Glu + Mdha + H – H_2_O]; 213 [Glu + Mdha + H]; 135 Adda fragment, Figure S6: Enhanced product ion mass spectrum of microcystin *m/z* 1054/528, Figure S7: Enhanced product ion mass spectrum of microcystin with the suggested structure MC-(H_4_)YR (*m/z* 1049) and the following fragment ions: *m/z* 978 [M + H – Ala]; 965 [M + H – Mdha]; 920 [M + H – Glu/Masp]; 893 [M + H – Arg]; 753 [Arg + Adda + Glu + Mdha + Ala + H]; 728 [Masp + Arg + Adda + Glu + H]; 725 [Arg + Adda + Glu + Mdha + Ala + H – CO]; 710 [Masp + Arg + Adda + Glu + H – H_2_O]; 682 [Arg + Adda + Glu + Mdha + H]; 624 [Mdha + Ala + (H_4_)Tyr + Masp + Arg + NH_3_ + H]; 607 [Mdha + Ala + (H_4_)Tyr + Masp + Arg + H]; 599 [Arg + Adda + Glu + H], 589 [M + H – (Adda + Glu) – H_2_O]; 582 [Arg + Adda + Glu + H – NH_3_]; 571 [Arg + Adda + Glu + H – CO], 523 [Ala + (H_4_)Tyr + Masp + Arg + H]; 495 [Ala + (H_4_)Tyr + Masp + Arg + H – CO]; 470 [Arg + Adda + H]; 446 [C_11_H_14_O + Glu + Mdha + Ala + H]; 418 [C_11_H_14_O + Glu + Mdha + Ala + H – CO]; 375 [C_11_H_14_O + Glu + Mdha + H]; 347 [C_11_H_14_O + Glu + Mdha + H – CO]; 284 [Glu + Mdha + Ala + H]; 213 [Glu + Mdha + H]; 195 [Glu + Mdha + H – H_2_O]; 163 [C_11_H_14_O + H]; 155 [Mdha + Ala + H]; 135 Adda fragment; 127 [Mdha + Ala + H – CO], Figure S8: Enhanced product ion mass spectrum of microcystin with the suggested structure MC-YR (*m/z* 1045) and the following fragment ions: *m/z* 974 [M + H – Ala]; 962 [M + H – Mdha]; 916 [M + H – Glu/Masp]; 889 [M + H – Arg]; 834 [M + H – (Glu + Mdha)]; 760 [M + H – (Masp + Arg)]; 683 [Masp + Arg + Adda + Glu + H – NH_3_ – CO]; 603 [M + H – (Adda + Glu)]; 599 [Arg + Adda + Glu + H], 576 [Glu + Mdha + Ala + Tyr + Masp + H]; 571 [Arg + Adda + Glu + H – CO]; 520 [Ala + Tyr + Masp + Arg + H]; 470 [Arg + Adda + H]; 448 [Tyr + Masp + Arg + H]; 432 [Tyr + Masp + Arg + H – NH_3_]; 375 [C_11_H_14_O + Glu + Mdha + H]; 347 [Adda fragmen + Glu + Mdha + H – NH_3_ – CO]; 303 [Masp + Arg – NH_3_]; 269 [Masp + Arg + H – NH_3_]; 213 [Glu + Mdha + H]; 195 [Glu + Mdha + H – H_2_O]; 163 [C_11_H_14_O + H]; 135 Adda fragment, 127 [Mdha + Ala + H – CO], Figure S9: Enhanced product ion mass spectrum of microcystin with the suggested structure MC-HphR (*m/z* 1043) and the following fragment ions: *m/z* 972 [M + H – Ala]; 909 [M + H – Adda fragment]; 892 [C_11_H_14_O + Glu + Mdha + Ala + Hph + MeAsp + Arg + H]; 887 [M + H – Arg]; 882 [M + H – Hph]; 831 [M + H – (Glu + Mdha)]; 728 [Masp + Arg + Adda + Glu + H]; 682 [Arg + Adda + Glu + Mdha + H]; 602 [M + H – (Adda + Glu)]; 599 [Arg + Adda + Glu + H]; 574 [M + H – (Adda + Arg)]; 571 [Arg + Adda + Glu + H – CO]; 518 [Ala + Hph + Masp + Arg + H]; 470 [Arg + Adda + H]; 446 [C_11_H_14_O + Glu + Mdha + Ala + H]; 375 [C_11_H_14_O + Glu + Mdha + H]; 347 [C_11_H_14_O + Glu + Mdha + H – CO]; 213 [Glu + Mdha + H]; 195 [Glu + Mdha + H – H_2_O]; 163 [C_11_H_14_O + H]; 135 Adda fragment; 127 [Mdha + Ala + H – CO], Figure S10: Enhanced product ion mass spectrum of microcystin with the suggested structure MC-RR (*m/z* 1038/519) and the following fragment ions: *m/z* 910 [M + H – Glu/Masp]; 887 [M + H – Arg]; 884 [M + H – (Mdha + Ala)]; 826 [M + H – (Glu + Mdha)]; 811 [M + H – (Ala + Arg)]; 755 [M + H – (Glu + Mdha + Ala)]; 731 [C_11_H_14_O + Glu + Mdha + Ala + Arg + Masp + H]; 702 [C_11_H_14_O + Glu + Mdha + Ala + Arg + Masp + H – CO]; 599 [Arg + Adda + Glu + H]; 579 [Adda + Glu + Mdha + Ala + H – H_2_O]; 571 [Arg + Adda + Glu + H – CO]; 470 [Arg + Adda + H]; 440 [Glu + Mdha + Ala + Arg + H]; 375 [C_11_H_14_O + Glu + Mdha + H]; 311 [Mdha + Ala + Arg + H]; 285 [Glu + Mdha + Ala + H]; 213 [Glu + Mdha + H]; 195 [Glu + Mdha + H – H_2_O]; 163 [C_11_H_14_O + H]; 135 Adda fragment; 127 [Mdha + Ala + H – CO], Figure S11: Enhanced product ion mass spectrum of microcystin with the suggested structure [Asp^3^]MC-RY (*m/z* 1031/516) and the following fragment ions: *m/z* 960 [M + H – Ala]; 916 [M + H – Asp]; 897 [M + H – Adda fragment]; 880 [C_11_H_14_O + Glu + Mdha + Ala + Arg + Asp + Tyr + H]; 868 [M + H – Tyr]; 819 [M + H – (Glu + Mdha)]; 760 [M + H – (Arg + Asp)]; 753 [M + H – (Tyr + Asp)]; 735 [M + H – (Tyr + Asp) – H_2_O]; 717 [C_11_H_14_O + Glu + Mdha + Ala + Arg + Asp + H]; 606 [Tyr + Adda + Glu + H]; 602 [C_11_H_14_O + Glu + Mdha + Ala + Arg + H]; 589 [Mdha + Ala + Arg + Asp + Tyr + H]; 555 [M + H – (Adda + Glu)]; 435 [Arg + Asp + Tyr + H]; 426 [Mdha + Ala + Arg + Asp + H]; 375 [C_11_H_14_O + Glu + Mdha + H]; 343 [Ala + Arg + Asp + H]; 311 [Mdha + Ala + Arg + H]; 213 [Glu + Mdha + H]; 163 [C_11_H_14_O + H]; 135 Adda fragment, Figure S12: Enhanced product ion mass spectrum of microcystin with the suggested structure [Asp^3^]MC-YR or [Asp^3^]MC-M(O_2_)R (*m/z* 1031/516) and the following fragment ions: *m/z* 960 [M + H – Ala]; 902 [M + H – Glu/Masp]; 897 [M + H – Adda fragment]; 880 [C_11_H_14_O + Glu + Mdha + Ala + Tyr/M(O_2_) + Asp + Arg + H]; 868 [M + H – Tyr/M(O_2_)]; 599 [Arg + Adda + Glu + H]; 589 [Mdha + Ala + Tyr/M(O_2_) + Asp + Arg + H]; 585 [Asp + Arg + Adda + H]; 562 [Glu + Mdha + Ala + Tyr/M(O_2_) + Asp + H]; 506 [Ala + Tyr/M(O_2_) + Asp + Arg + H]; 470 [Arg + Adda + H]; 446 [C_11_H_14_O + Glu + Mdha + Ala + H]; 435 [Tyr/M(O_2_) + Asp + Arg + H]; 375 [C_11_H_14_O + Glu + Mdha + H]; 357 [C_11_H_14_O + Glu + Mdha + H – H_2_O]; 347 [C_11_H_14_O + Glu + Mdha + H – CO]; 318 [Mdha + Ala + Tyr/M(O_2_) + H]; 213 [Glu + MeDha + H]; 135 Adda fragment; 127 [Mdha + Ala + H – CO], Figure S13: Enhanced product ion mass spectrum of microcystin with the suggested structure MC-FR (*m/z* 1029) and the following fragment ions: *m/z* 957 [M + H – Ala]; 946 [M + H – Mdha]; 900 [M + H – Glu/Masp]; 895 [M + H – Adda fragment]; 878 [M + H – Glu/Masp – H_2_O]; 817 [M + H – (Glu + Mdha)]; 728 [Masp + Arg + Adda + Glu + H]; 682 [Arg + Adda + Glu + Mdha + H]; 604 [M + H – (Adda + Glu) + NH_3_]; 599 [Arg + Adda + Glu + H]; 587 [M + H – (Adda + Glu)]; 571 [Arg + Adda + Glu + H – CO]; 560 [M + H – (Arg + Adda)]; 504 [Ala + Phe + Masp + Arg + H]; 470 [Arg + Adda + H]; 433 [Phe + Masp + Arg + H]; 431 [Mdha + Ala + Phe + Masp + H]; 375 [C_11_H_14_O + Glu + Mdha + H]; 356 [Ala + Phe + Masp + H]; 348 [Ala + Phe + Masp + H]; 302 [Mdha + Ala + Phe + H]; 284 [Glu + Mdha + Ala + H]; 213 [Glu + Mdha + H]; 195 [Glu + Mdha + H – H_2_O]; 163 [C_11_H_14_O + H]; 135 Adda fragment; 127 [Mdha + Ala + H – CO]; 120 Phe immonium ion, Figure S14: Enhanced product ion mass spectrum of microcystin with the suggested structure MC-LW (*m/z* 1025) and the following fragment ions: *m/z* 976 [M + H – CH_3_OH – NH_3_]; 925 [M + H – Mdha – NH_3_]; 891 [M + H – Adda fragment]; 873 [C_11_H_14_O + Glu + Mdha + Ala + Leu + Masp + Trp + H]; 693 [Adda + Glu + Mdha + Ala + Leu + H – NH_3_]; 583 [M + H – (Adda + Glu)]; 580 [Adda + Glu + Mdha + Ala + H – NH_3_]; 565 [Mdha + Ala + Leu + Masp + Trp + H – H_2_O]; 559 [C_11_H_14_O + Glu + Mdha + Ala + Leu + H]; 555 [Mdha + Ala + Leu + Masp + Trp + H – CO]; 509 [Adda + Glu + Mdha + H – NH_3_]; 500 [Ala + Leu + Masp + Trp + H]; 472 [Ala + Leu + Masp + Trp + H – CO]; 446 [C_11_H_14_O + Glu + Mdha + Ala + H]; 429 [Leu + Masp + Trp + H]; 397 [Mdha + Ala + Leu + Masp + H]; 375 [C_11_H_14_O + Glu + Mdha + H]; 347 [C_11_H_14_O + Glu + Mdha + H – CO]; 316 [Trp + Masp + H]; 298 [Trp + Masp + H – H_2_O]; 288 [Trp + Masp + H – CO]; 213 [Glu + Mdha + H]; 195 [Glu + Mdha + H – H_2_O]; 187 [Trp + H]; 163 [C_11_H_14_O + H]; 159 Trp immonium ion; 135 Adda fragment; 127 [Mdha + Ala + H – CO], Figure S15: Enhanced product ion mass spectrum of microcystin with the suggested structure [Dha^7^]MC-RR (*m/z* 1024/512) and the following fragment ions: *m/z* 890 [M + H – Adda fragment]; 872 [M + H – Adda fragment – H_2_O]; 717 [C_11_H_14_O + Glu + Dha + Ala + Arg + Masp + H]; 668 [Arg + Adda + Glu + Dha + H]; 588 [C_11_H_14_O + Glu + Dha + Ala + Arg + H]; 582 [Dha + Ala + Arg + Masp + Arg + H]; 565 [Dha + Ala + Arg + Masp + Arg + H – NH_3_]; 426 [Glu + Dha + Ala + Arg + H]; 297 [Dha + Ala + Arg + H]; 269 [Dha + Ala + Arg + H – CO]; 199 [Glu + Dha + H]; 181 [Glu + Dha + H – H_2_O]; 141 [Dha + Ala + H]; 135 Adda fragment; 113 [Dha + Ala + H – CO], Figure S16: Enhanced product ion mass spectrum of microcystin with the suggested structure MC-HilR (*m/z* 1009) and the following fragment ions: *m/z* 992 [M + H – NH_3_]; 938 [M + H – Ala]; 926 [M + H – Mdha]; 880 [M + H – Glu/Masp]; 875 [M + H – Adda fragment]; 858 [C_11_H_14_O + Glu + Mdha + Ala + Hil + Masp + Arg + H]; 853 [M + H – Arg]; 797 [M + H - (Glu + Mdha)]; 753 [M + H – (Hil + Masp); 728 [Masp + Arg + Adda + Glu + H]; 682 [Arg + Adda + Glu + Mdha + H]; 599 [Arg + Adda + Glu + H]; 584 [Mdha + Ala + Hil + Masp + Arg + H + NH_3_]; 573 [C_11_H_14_O + Glu + Mdha + Ala + Hil + H]; 567 [M + H – (Adda + Glu)]; 540 [Glu + Mdha + Ala + Hil + Masp + H]; 484 [Ala + Hil + Masp + Arg + H]; 470 [Arg + Adda + H]; 446 [C_11_H_14_O + Glu + Mdha + Ala + H]; 412 [Hil + Masp + Arg + H]; 396 [Hil + Masp + Arg + H – NH_3_]; 375 [C_11_H_14_O + Glu + Mdha + H]; 347 [C_11_H_14_O + Glu + Mdha + H – CO]; 213 [Glu + Mdha + H]; 195 [Glu + Mdha + H – H_2_O]; 163 [C_11_H_14_O + H]; 135 Adda fragment; 127 [Mdha + Ala + H – CO], Figure S17: Enhanced product ion mass spectrum of microcystin with the suggested structure MC-LY (*m/z* 1002) and the following fragment ions: *m/z* 985 [M + H – NH_3_]; 902 [M + H – Mdha – NH_3_]; 868 [M + H – Adda fragment]; 851 [C_11_H_14_O + Glu + Mdha + Ala + Leu + Masp + Tyr + H]; 818 [M + H – (Ala + Leu)]; 693 [Adda + Glu + Mdha + Ala + Leu + H – NH_3_]; 580 [Adda + Glu + Mdha + Ala + H – NH_3_]; 560 [M + H – (Adda + Glu)]; 559 [C_11_H_14_O + Glu + Mdha + Ala + Leu + H]; 509 [Adda + Glu + Mdha + H – NH_3_]; 477 [Tyr + Adda + H]; 446 [C_11_H_14_O + Glu + Mdha + Ala + H]; 406 [Leu + Masp + Tyr + H]; 397 [Mdha + Ala + Leu + Masp + H]; 375 [C_11_H_14_O + Glu + Mdha + H]; 347 [C_11_H_14_O + Glu + Mdha + H – CO]; 293 [Masp + Tyr + H]; 213 [Glu + Mdha + H]; 195 [Glu + Mdha + H – H_2_O]; 163 [C_11_H_14_O + H]; 155 [Mdha + Ala + H]; 136 Tyr immonium ion; 135 Adda fragment; 127 [Mdha + Ala + H – CO]; 86 Ile immonium ion, Figure S18: Enhanced product ion mass spectrum of microcystin with the suggested structure MC-LR (*m/z* 995) and the following fragment ions: *m/z* 977 [M + H – H_2_O]; 967 [M + H – CO]; 924 [M + H – Ala]; 912 [M + H – Mdha]; 866 [M + H – Glu/Masp]; 839 [M + H – Arg]; 783 [M + H – (Glu + Mdha)]; 728 [Masp + Arg + Adda + Glu + H]; 712 [M + H – (Glu + Mdha + Ala)]; 682 [M + H – (Ala + Leu + Masp)]; 599 [Arg + Adda + Glu + H]; 571 [Arg + Adda + Glu + H – CO]; 553 [M + H – (Adda + Glu)]; 526 [Adda + Glu + Mdha + H]; 470 [Arg + Adda + H]; 453 [Ala + Leu + Masp + Arg + H – NH_3_]; 397 [Glu + Mdha + Ala + Leu + H]; 375 [C_11_H_14_O + Glu + Mdha + H]; 347 [C_11_H_14_O + Glu + Mdha + H – CO]; 269 [Masp + Arg + H – NH_3_]; 213 [Glu + Mdha + H]; 195 [Glu + Mdha + H – H_2_O]; 163 [C_11_H_14_O + H]; 135 Adda fragment, Figure S19: Enhanced product ion mass spectrum of microcystin with the suggested structure [Asp^3^]MC-LY (*m/z* 988) and the following fragment ions: *m/z* 854 [M + H – Adda fragment]; 837 [C_11_H_14_O + Glu + Mdha + Ala + Leu + Asp + Tyr + H]; 580 [Adda + Glu + Mdha + Ala + H – NH_3_]; 546 [M + H – (Adda + Glu)]; 477 [Tyr + Adda + H]; 463 [Ala + Leu + Asp + Tyr + H]; 446 [C_11_H_14_O + Glu + Mdha + Ala + H]; 383 [Mdha + Ala + Leu + Asp + H]; 375 [C_11_H_14_O + Glu + Mdha + H]; 266 [Glu + Mdha + Ala + H – H_2_O]; 251 [Asp + Tyr + H – CO]; 213 [Glu + Mdha + H]; 195 [Glu + Mdha + H – H_2_O]; 163 [C_11_H_14_O + H]; 135 Adda fragment, Figure S20: Enhanced product ion mass spectrum of microcystin with the suggested structure MC-LF (*m/z* 986) and the following fragment ions: *m/z* 969 [M + H – NH_3_]; 857 [M + H – Glu/Masp]; 852 [M + H – Adda fragment]; 802 [M + H – (Ala + Leu)]; 693 [Adda + Glu + Mdha + Ala + Leu + H – NH_3_]; 580 [Adda + Glu + Mdha + Ala + H – NH_3_]; 559 [C_11_H_14_O + Glu + Mdha + Ala + Leu + H]; 544 [M + H – (Adda + Glu)]; 509 [Glu + Mdha + Ala + Leu + Masp + H – NH_3_]; 461 [Ala + Leu + Masp + Phe + H]; 446 [C_11_H_14_O + Glu + Mdha + Ala + H]; 397 [Glu + Mdha + Ala + Leu + H]; 390 [Leu + Masp + Phe + H]; 375 [C_11_H_14_O + Glu + Mdha + H]; 347 [C_11_H_14_O + Glu + Mdha + H – CO]; 249 [Masp + Phe + H – CO]; 213 [Glu + Mdha + H]; 195 [Glu + Mdha + H – H_2_O]; 163 [C_11_H_14_O + H]; 135 Adda fragment; 127 [Mdha + Ala + H – CO], Figure S21: Enhanced product ion mass spectrum of microcystin with the suggested structure [Asp^3^]MCLR (*m/z* 981) and the following fragment ions: *m/z* 910 [M + H – Ala]; 847 [M + H – Adda fragment]; 830 [C_11_H_14_O + Glu + Mdha + Ala + Leu + Asp + Arg + H]; 753 [Arg + Adda + Glu + Mdha + H]; 668 [M + H – Adda]; 599 [Arg + Adda + Glu + H]; 571 [Arg + Adda + Glu + H – CO]; 539 [M + H – (Adda + Glu)]; 470 [Arg + Adda + H]; 456 [M + H – (Adda + Glu + Mdha)]; 446 [C_11_H_14_O + Glu + Mdha + Ala + H]; 383 [Mdha + Ala + Leu + Asp + H]; 375 [C_11_H_14_O + Glu + Mdha + H]; 369 [Glu + Mdha + Ala + Leu + H – CO]; 347 [C_11_H_14_O + Glu + Mdha + H – CO]; 213 [Glu + Mdha + H]; 195 [Glu + Mdha + H – H_2_O]; 163 [C_11_H_14_O+H]; 135 Adda fragment; 105 Dha, Figure S22: Enhanced product ion mass spectrum of microcystin with the suggested structure [Dha^7^]MCLR (*m/z* 981) and the following fragment ions: *m/z* 910 [M + H – Ala]; 852 [M + H – Glu/Mdha]; 830 [C_11_H_14_O + Glu + Dha + Ala + Leu + Masp + Arg + H]; 825 [M + H – Arg]; 728 [Masp + Arg + Adda + Glu + H]; 712 [Leu + Masp + Arg + Adda + H]; 599 [Arg + Adda + Glu + H]; 583 [Adda + Glu + Dha + Ala + H]; 571 [Arg + Adda + Glu + H – CO]; 539 [M + H – (Adda + Glu)]; 512 [Adda + Glu + Dha + H]; 470 [Arg + Adda + H]; 432 [C_11_H_14_O + Glu + Dha + Ala + H]; 399 [Leu + Masp + Arg + H]; 383 [Glu + Dha + Ala + Leu + H]; 361 [C_11_H_14_O + Glu + Dha + H]; 334 [C_11_H_14_O + Glu + Dha + H – CO]; 269 [Glu + Dha + Ala + H]; 181 [Glu + Dha + H – H_2_O]; 163 [C_11_H_14_O + H]; 141 [Dha + Ala + H]; 135 Adda fragment.
