# Supplementary material for: Characterization and Diversity of Microcystins Produced by Cyanobacteria from the Curonian Lagoon (SE Baltic Sea)
Source: Toxins (Basel). 2021 Nov 24;13(12):838. doi: 10.3390/toxins13120838 (PMC8703916; doi:10.3390/toxins13120838)
Supplement: Supplementary file 1 [file toxins-13-00838-s001.zip › toxins-1480067-supplementary.pdf]

# Supplementary Materials: Characterization and Diversity of Microcystins Produced by Cyanobacteria from the Curonian Lagoon (SE Baltic Sea)

**Donata Overlingė, Anna Toruńska-Sitarz, Marija Kataržytė, Renata Pilkaitytė, Greta Gyraitė and Hanna Mazur-Marzec**

**Table S1.** The abbreviations of amino acids and their full names.

| Abbreviation | Full Name |
|---|---|
| Adda | (*2S,3S,8S,9S*)-3-Amino-9-methoxy-2,6,8-trimethyl-10-phenyldeca-4,6-dienoic acid |
| (H4)Tyr | tetrahydrotyrosine |
| Arg | arginine |
| D-Ala | D-alanine |
| D-Asp | D-aspartate |
| D-Glu | D-glutamic acid |
| Dha | dehydroalanine |
| D-Masp | N-methyl-D-aspartate |
| D-Ser | D-serine |
| Har | homoarginine |
| Hil | homoisoleucine |
| Hph | homophenylalanine |
| Hty | homotyrosine |
| Leu | leucine |
| M(O2) or Met(O2) | methionine sulfone |
| Mdha | N-methyldehydroalanine |
| Phe | phenylalanine |
| Trp | tryptophan |
| Tyr | tyrosine |
| Tyr(OMe) | methyl tyrosinate |

**Table S2.** Presence and dominance of cyanobacteria species in the samples from the Curonian Lagoon. Cyanobacteria dominance was expressed as a percentage of total cyanobacteria biomass.

| **Samples** | **30 May 2018** | **13 Jun 2018** | **27 Jun 2018** | **11 Jul 2018** | **23 Jul 2018** | **3 Aug 2018** | **9 Aug 2018** | **16 Aug 2018** | **30 Aug 2018** | **19 Sep 2018** | **17 Oct 2019** | **3 Jul 2020** |
|---|---|---|---|---|---|---|---|---|---|---|---|---|
| **Total Phytoplankton Biomass, mg L$^{-1}$** | **8.85** | **12.72** | **12.11** | **19.76** | **39.72** | **23.9** | **19.07** | **15.99** | **16.71** | **33.29** | **14.62** | **4.10** |
| **Total Cyanobacteria Biomass, mg L$^{-1}$** | **1.17** | **7.88** | **3.22** | **2.61** | **1.06** | **3.41** | **2.78** | **2.05** | **6.66** | **12.39** | **8.86** | **2.65** |
| *Anabaena Cylindrica* | | | | | | | | | | | | |
| *Anabaena Mendotea* | | | | | | | | | | | | |
| *Anabaena Planctonica* | | | | | | | | | | | | |
| *Anabaena* sp. | | | | | | | | | | | | |
| *Anabaena Spiroides* | | | | | | | | | | | | |
| *Anathece Clatrata* | | | | | | | | | | | | |
| *Anathece Minutissima* | | | | | | | | | | | | |
| *Aphanizomenon Flosaquae* | | | | | | | | | | | | |
| *Aphanizomenon Gracile* | | | | | | | | | | | | |
| *Aphanocapsa Delicatissima* | | | | | | | | | | | | |
| *Aphanocapsa Holsatica* | | | | | | | | | | | | |
| *Aphanocapsa Incerta* | | | | | | | | | | | | |
| *Aphanocapsa Planctonica* | | | | | | | | | | | | |
| *Aphanocapsa* sp. | | | | | | | | | | | | |
| *Aphanocapsa Stagnalis* | | | | | | | | | | | | |
| *Aphanothece Minutissima* | | | | | | | | | | | | |
| *Aphanothece* sp. | | | | | | | | | | | | |
| *Chroococcus Aphanocapsoides* | | | | | | | | | | | | |
| *Chroococcus Minimus* | | | | | | | | | | | | |
| *Chroococcus Turgidus* | | | | | | | | | | | | |
| *Coelomoron Pussilum* | | | | | | | | | | | | |
| *Coelomoron Pussilum* | | | | | | | | | | | | |
| *Coelosphaerium Kuetzingianum* | | | | | | | | | | | | |
| *Cuspidothrix Issatschenkoi* | | | | | | | | | | | | |
| *Cyanodictyon Imperfectum* | | | | | | | | | | | | |
| *Cyanodictyon Planctonicum* | | | | | | | | | | | | |
| *Cyanodictyon Reticulatum* | | | | | | | | | | | | |
| *Cyanodictyon sp.* | | | | | | | | | | | | |
| *Dolichospermum Crassum* | | | | | | | | | | | | |
| *Dolichospermum Flosaquae* | | | | | | | | | | | | |
| *Dolichospermum Lemmermanii* | | | | | | | | | | | | |
| *Dolichospermum Planctonicum* | | | | | | | | | | | | |
| *Limnococcus Limneticus* | | | | | | | | | | | | |
| *Limnothrix Redekei* | | | | | | | | | | | | |
| *Merismopedia Punctata* | | | | | | | | | | | | |
| *Merismopedia* sp. | | | | | | | | | | | | |

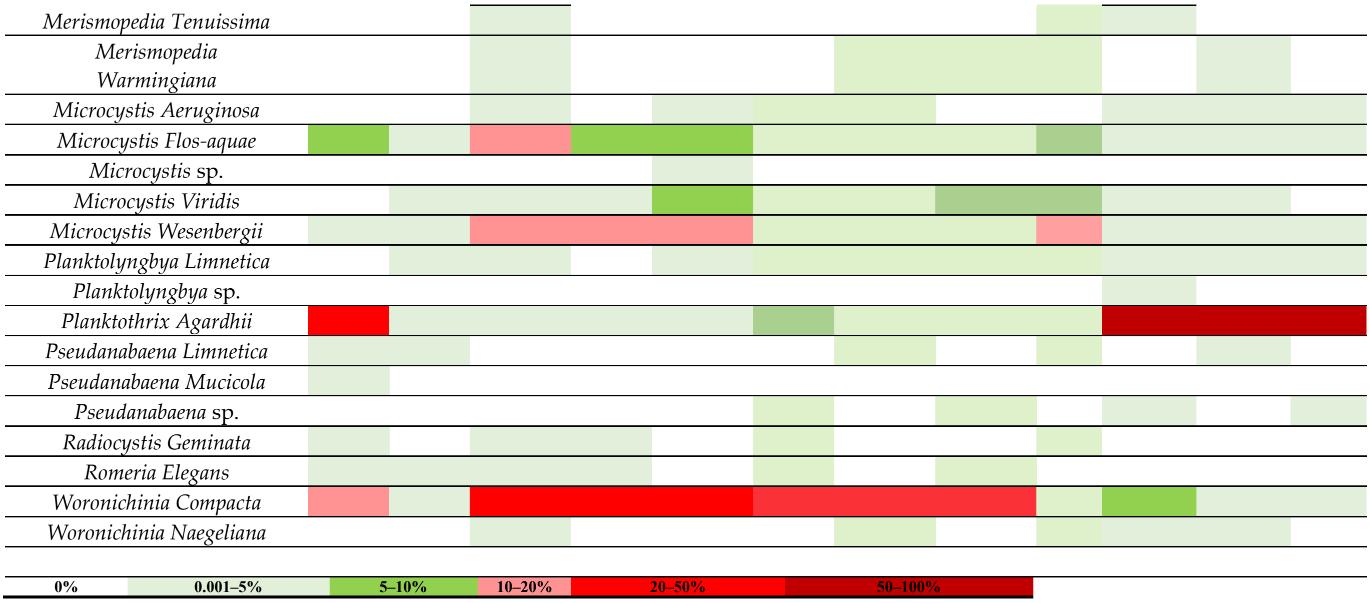


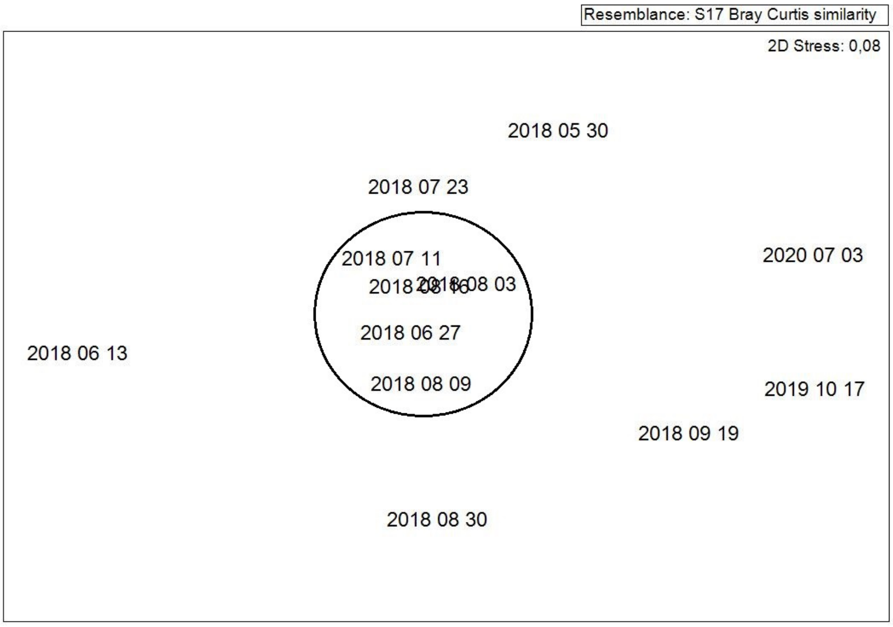


**Figure S1.** nMDS plot based on Bray-Curtis similarity matrix of dominating cyanobacteria species biomass. Only samples for which concentrations of microcystins were calculated in μg $L^{-1}$ were used for RDA analysis.

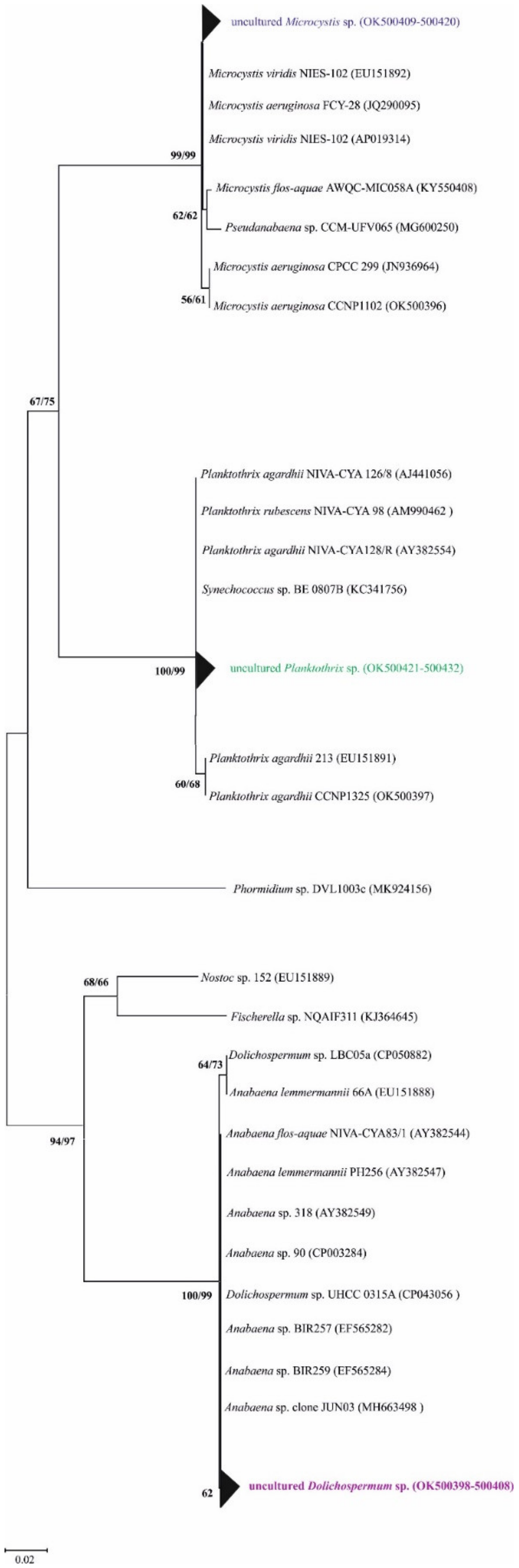


**Figure S2.** Phylogenetic relationships of sequences retrieved from the studied environmental samples (marked in colors), their closest relatives and representants of other potent microcystins producing genera, based on the alignment of partial (218 b) *mcyE* sequences. Numbers above branches indicate the bootstrap values for NJ and ML method. Accession numbers in NCBI are presented in brackets.

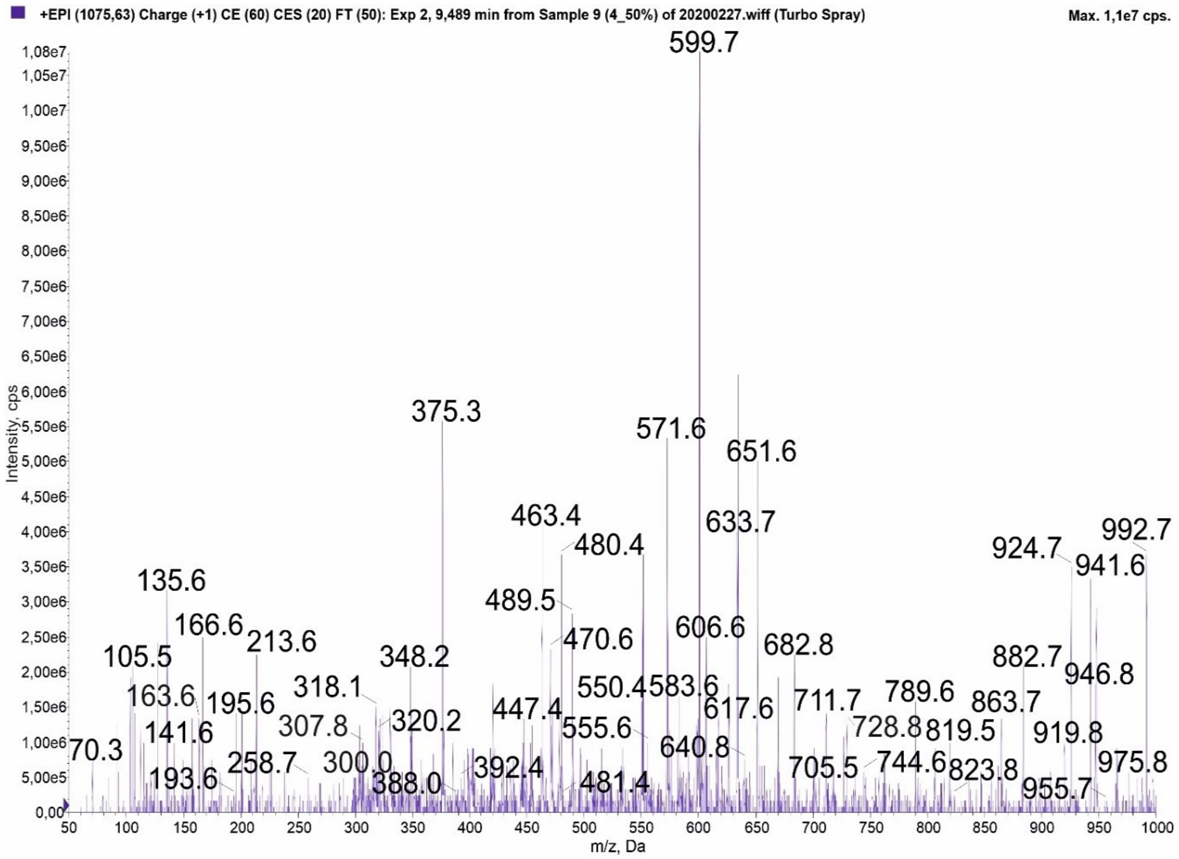


**Figure S3.** Enhanced product ion mass spectrum of microcystin with the suggested structure [Ser[1]]MC-HtyR (*m*/*z* 1075) and the following fragment ions: *m*/*z* 992 [M + H – Mdha]; 946 [M + H – Glu/Masp]; 941 [M + H – Adda fragment]; 924 [$C_{11}H_{14}O$ + Glu + Mdha + Ser + Hty + Masp + Arg + H]; 919 [M + H – Arg]; 882 [Masp + Arg + Adda + Glu + Mdha + H]; 863 [Ser + Hty + Masp + Arg + Adda + H]; 728 [Masp + Arg + Adda + Glu + H]; 682 [Arg + Adda + Glu + Mdha + H]; 633 [Mdha + Ser + Hty + Masp + Arg + H]; 606 [Glu + Mdha + Ser + Hty + Masp + H]; 599 [Arg + Adda + Glu + H], 571 [Arg + Adda + Glu + H – CO], 550 [Ser + Hty + Masp + Arg + H]; 470 [Arg + Adda + H]; 463 [$C_{11}H_{14}O$ + Glu + Mdha + Ser + H]/[Hty + Masp + Arg + H]; 375 [$C_{11}H_{14}O$ + Glu + Mdha + H]; 348 [$C_{11}H_{14}O$ + Glu + Mdha + H – CO]/[Mdha + Ser + Hty + H]; 307 [Hty + Masp + H]; 300 [Glu + Mdha + Ser + H]; 213 [Glu + Mdha + H]; 163 [$C_{11}H_{14}O$ + H]; 135 Adda fragment; or enhanced product ion mass spectrum of microcystin with the suggested structure MC-Y(OMe)R (*m*/*z* 1075) and the following fragment ions: *m*/*z* 992 [M + H – Mdha]; 946 [M + H – Glu/Masp]; 941 [M+H – Adda fragment]; 924 [$C_{11}H_{14}O$ + Glu + Mdha + Ala + Tyr(OMe) + Masp + Arg + H]; 919 [M + H – Arg]; 882 [Masp + Arg + Adda + Glu + Mdha + H]; 863 [Ala + Tyr(OMe) + Masp + Arg + Adda + H]; 728 [Masp + Arg + Adda + Glu + H]; 682 [Arg + Adda + Glu + Mdha + H]; 633 [Mdha + Ala + Tyr(OMe) + Masp + Arg + H]; 606 [Glu + Mdha + Ala + Tyr(OMe) + Masp + H]; 599 [Arg + Adda + Glu + H], 571 [Arg + Adda + Glu + H – CO], 550 [Ala + Tyr(OMe) + Masp + Arg + H]; 480 [Tyr(OMe) + Masp + Arg + H]; 470 [Arg + Adda + H]; 447 [$C_{11}H_{14}O$ + Glu + Mdha + Ala + H]; 375 [$C_{11}H_{14}O$ + Glu + Mdha + H]; 348 [$C_{11}H_{14}O$ + Glu + Mdha + H – CO]/[Mdha + Ala + Tyr(OMe) + H]; 213 [Glu + Mdha + H]; 163 [$C_{11}H_{14}O$ + H]; 135 Adda fragment.

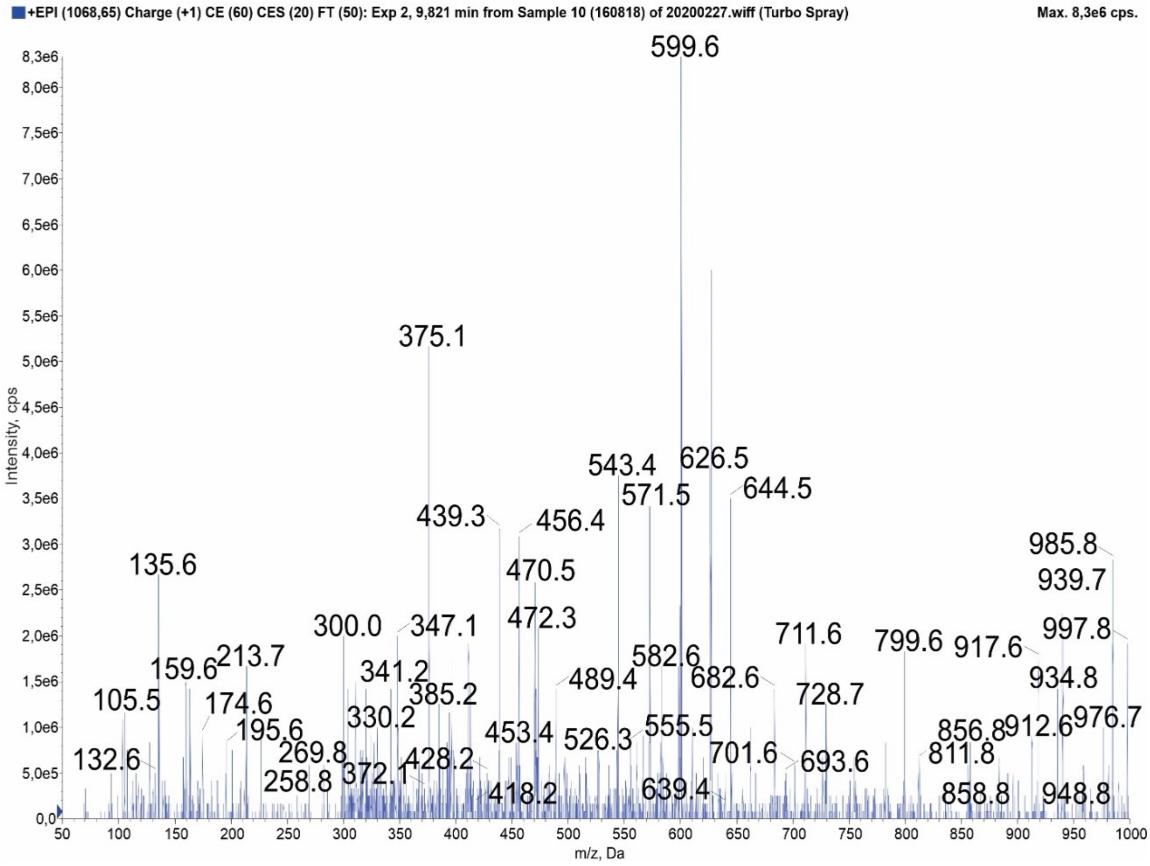


**Figure S4.** Enhanced product ion mass spectrum of microcystin with the suggested structure MC-WR (*m*/*z* 1068) and the following fragment ions: *m*/*z* 997 [M + H – Ala]; 985 [M + H – Mdha]; 939 [M + H – Glu/Masp]; 934 [M + H – Adda fragment]; 917 [M + H – Adda fragment – $H_2O$]; 912 [M + H – Adda]; 856 [M + H – (Glu + Mdha)]; 811 [M + H – (Ala + Trp)]; 728 [Masp + Arg + Adda + Glu + H]; 682 [Arg + Adda + Glu + Mdha + H]; 626 [Mdha + Ala + Trp + Masp + Arg + H]; 599 [Arg + Adda + Glu + H], 582 [Agr + Adda + Glu + H – $NH_3$]; 571 [Masp + Arg + Adda + H – CO]/[Arg + Adda + Glu + H – CO]; 543 [Ala + Trp + Masp + Arg + H]; 526 [Adda + Glu + Mdha + H]; 472 [Trp + Masp + Arg + H]; 470 [Arg + Adda + H]; 456 [Trp + Masp + Arg + H – $NH_3$]; 375 [$C_{11}H_{14}O$ + Glu + Mdha + H]; 347 [$C_{11}H_{14}O$ + Glu + Mdha + H – CO]; 341 [Mdha + Ala + Trp + H]; 269 [Masp + Arg + H – $NH_3$]; 258 [Ala + Trp + H]; 213 [Glu + Mdha + H]; 195 [Glu + Mdha + H – $H_2O$]; 135 Adda fragment; or enhanced product ion mass spectrum of microcystin with the suggested structure [Ser$^{1}$]MC-HarR (*m*/*z* 1068) and the following fragment ions: *m*/*z* 997 [M + H – Ala]; 985 [M + H – Mdha]; 939 [M + H – Glu/Masp]; 934 [M + H – Adda fragment]; 917 [M + H – Adda fragment – $H_2O$]; 912 [M + H – Adda]; 856 [M + H – (Glu + Mdha)]; 811 [M + H – (Ser + Har)]; 728 [Masp + Arg + Adda + Glu + H]; 682 [Arg + Adda + Glu + Mdha + H]; 626 [Mdha + Ser + Har + Masp + Arg + H]; 599 [Arg + Adda + Glu + H], 582 [Agr + Adda + Glu + H – $NH_3$]; 571 [Masp + Arg + Adda + H – CO]/[Arg + Adda + Glu + H – CO]; 543 [Ser + Har + Masp + Arg + H]; 526 [Adda + Glu + Mdha + H]; 470 [Arg + Adda + H]; 456 [Har + Masp + Arg + H]; 375 [$C_{11}H_{14}O$ + Glu + Mdha + H]; 347 [$C_{11}H_{14}O$ + Glu + Mdha + H – CO]; 341 [Mdha + Ser + Har + H]; 300 [Har + Masp + H]/[Glu + Mdha + Ser + H]; 258 [Ser + Har + H]; 213 [Glu + Mdha + H]; 195 [Glu + Mdha + H – $H_2O$]; 135 Adda fragment.

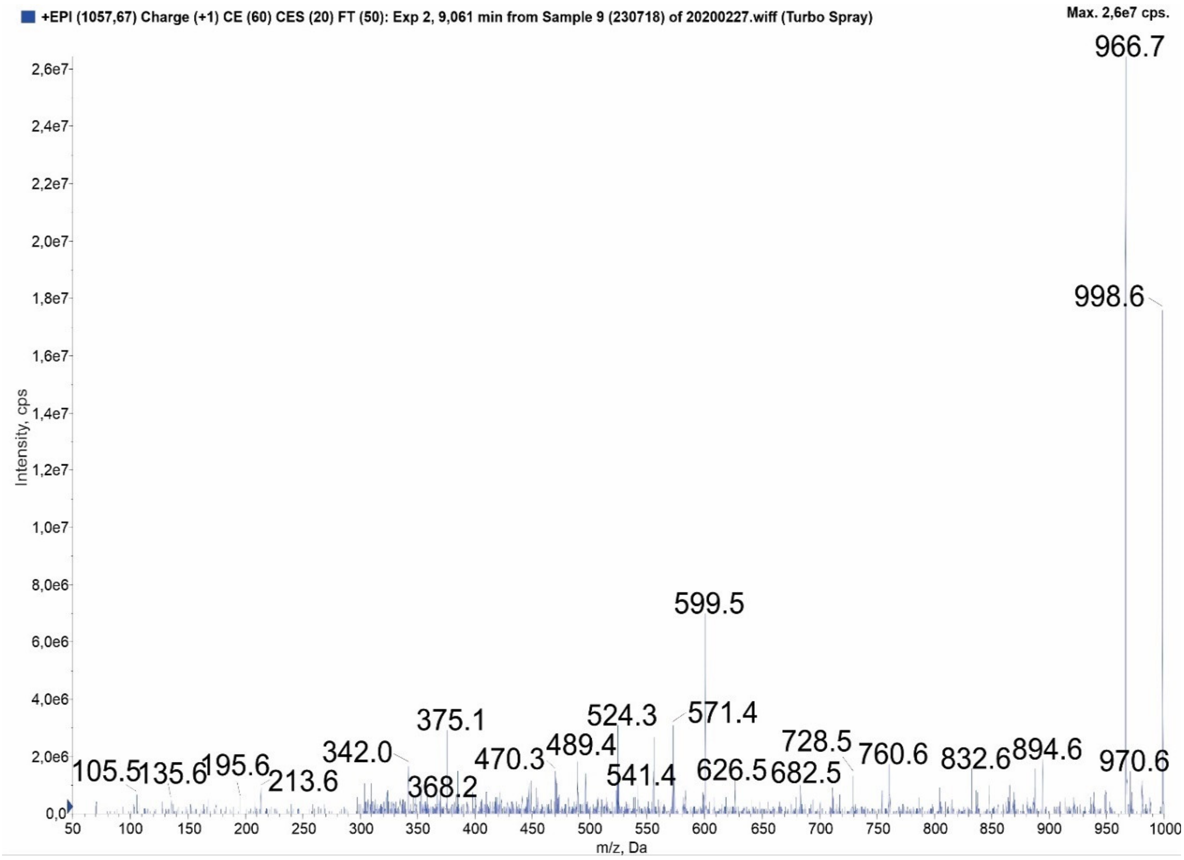


**Figure S5.** Enhanced product ion mass spectrum of microcystin with the partially elucidated structure MC-XR (*m*/*z* 1057) and the following fragment ions: 728 [Masp + Arg + Adda + Glu + H]; 682 [Arg + Adda + Glu + Mdha + H]; 599 [Arg + Adda + Glu + H], 571 [Arg + Adda + Glu + H – CO], 470 [Arg + Adda + H]; 375 [$C_{11}H_{14}O$ + Glu + Mdha + H]; 195 [Glu + Mdha + H – $H_2O$]; 213 [Glu + Mdha + H]; 135 Adda fragment.

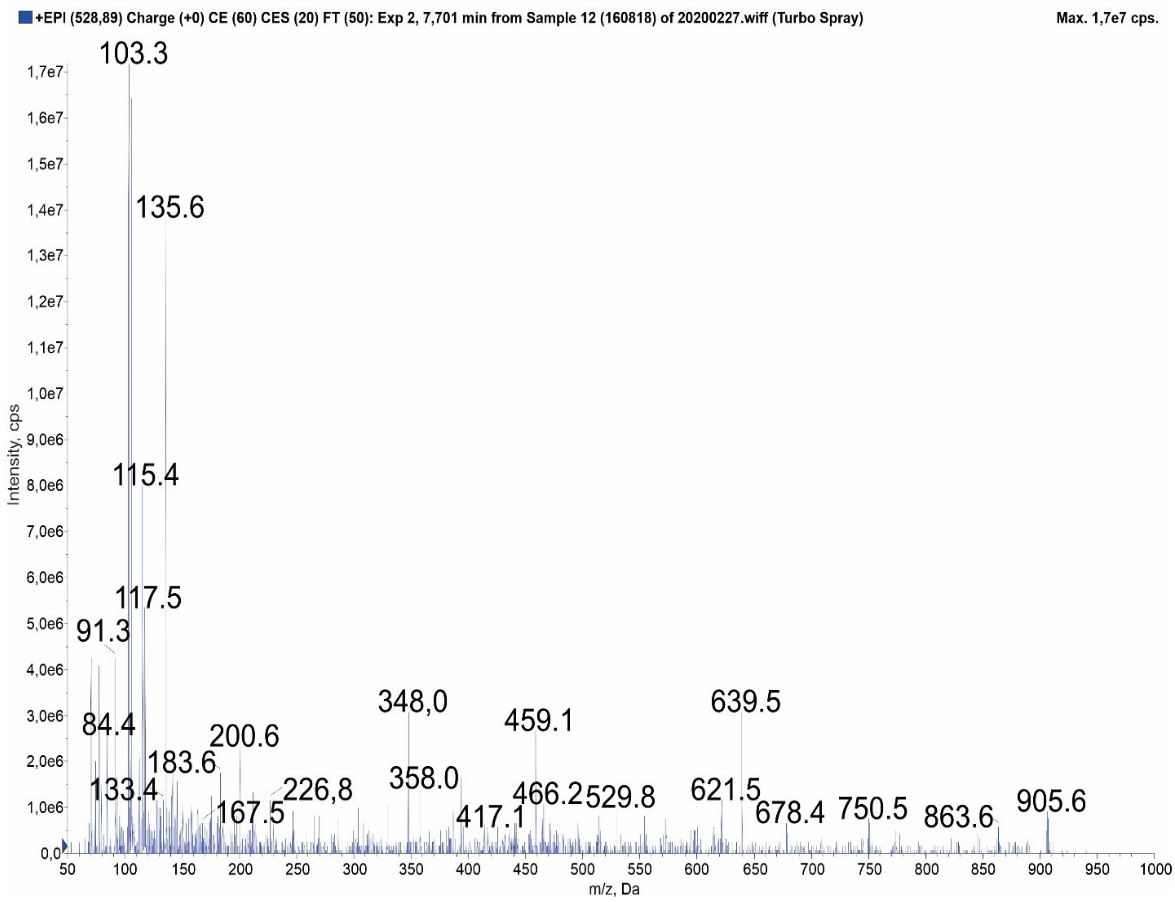


**Figure S6.** Enhanced product ion mass spectrum of microcystin *m*/*z* 1054/528.

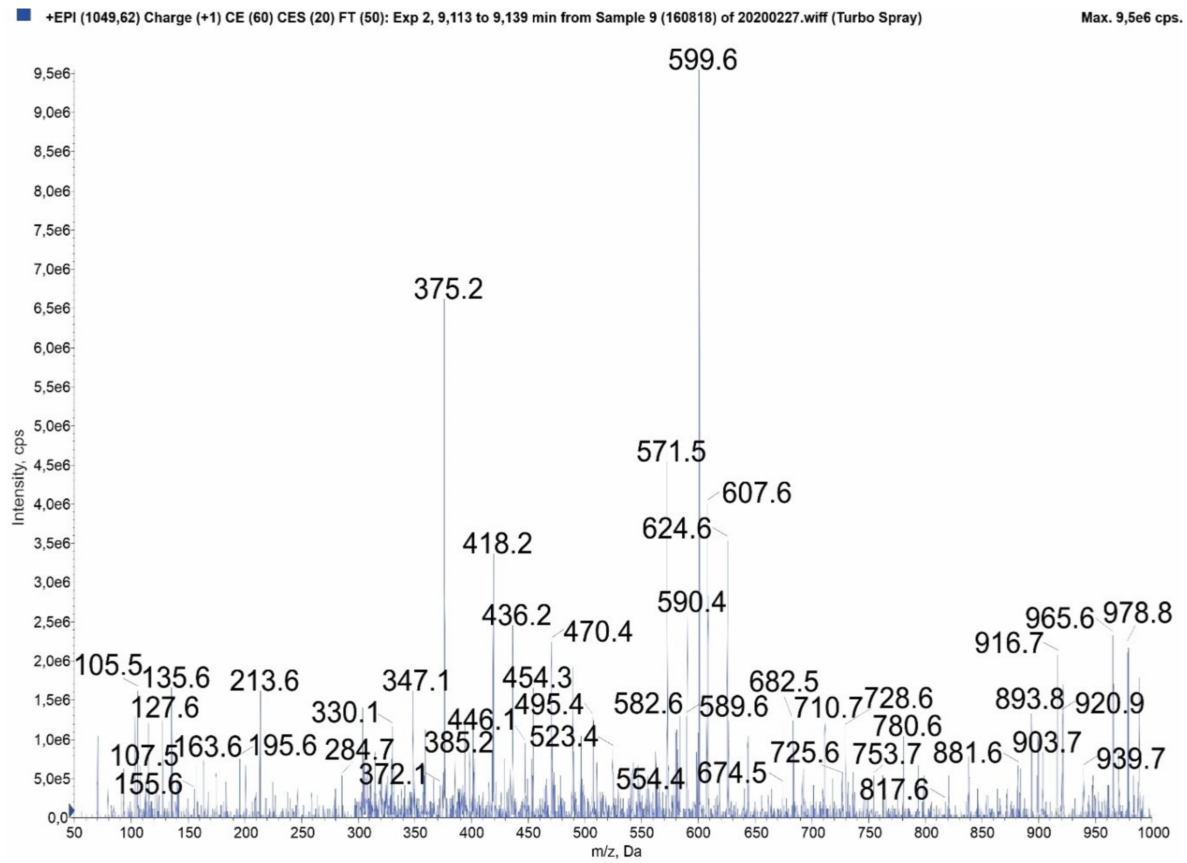


**Figure S7.** Enhanced product ion mass spectrum of microcystin with the suggested structure MC-($H_4$)YR (*m*/*z* 1049) and the following fragment ions: *m*/*z* 978 [M + H – Ala]; 965 [M + H – Mdha]; 920 [M + H – Glu/Masp]; 893 [M + H – Arg]; 753 [Arg + Adda + Glu + Mdha + Ala + H]; 728 [Masp + Arg + Adda + Glu + H]; 725 [Arg + Adda + Glu + Mdha + Ala + H – CO]; 710 [Masp + Arg + Adda + Glu + H – $H_2O$]; 682 [Arg + Adda + Glu + Mdha + H]; 624 [Mdha + Ala + ($H_4$)Tyr + Masp + Arg + $NH_3$ + H]; 607 [Mdha + Ala + ($H_4$)Tyr + Masp + Arg + H]; 599 [Arg + Adda + Glu + H], 589 [M + H – (Adda + Glu) – $H_2O$]; 582 [Arg + Adda + Glu + H – $NH_3$]; 571 [Arg + Adda + Glu + H – CO], 523 [Ala + ($H_4$)Tyr + Masp + Arg + H]; 495 [Ala + ($H_4$)Tyr + Masp + Arg + H – CO]; 470 [Arg + Adda + H]; 446 [$C_{11}H_{14}O$ + Glu + Mdha + Ala + H]; 418 [$C_{11}H_{14}O$ + Glu + Mdha + Ala + H – CO]; 375 [$C_{11}H_{14}O$ + Glu + Mdha + H]; 347 [$C_{11}H_{14}O$ + Glu + Mdha + H – CO]; 284 [Glu + Mdha + Ala + H]; 213 [Glu + Mdha + H]; 195 [Glu + Mdha + H – $H_2O$]; 163 [$C_{11}H_{14}O$ + H]; 155 [Mdha + Ala + H]; 135 Adda fragment; 127 [Mdha + Ala + H – CO].

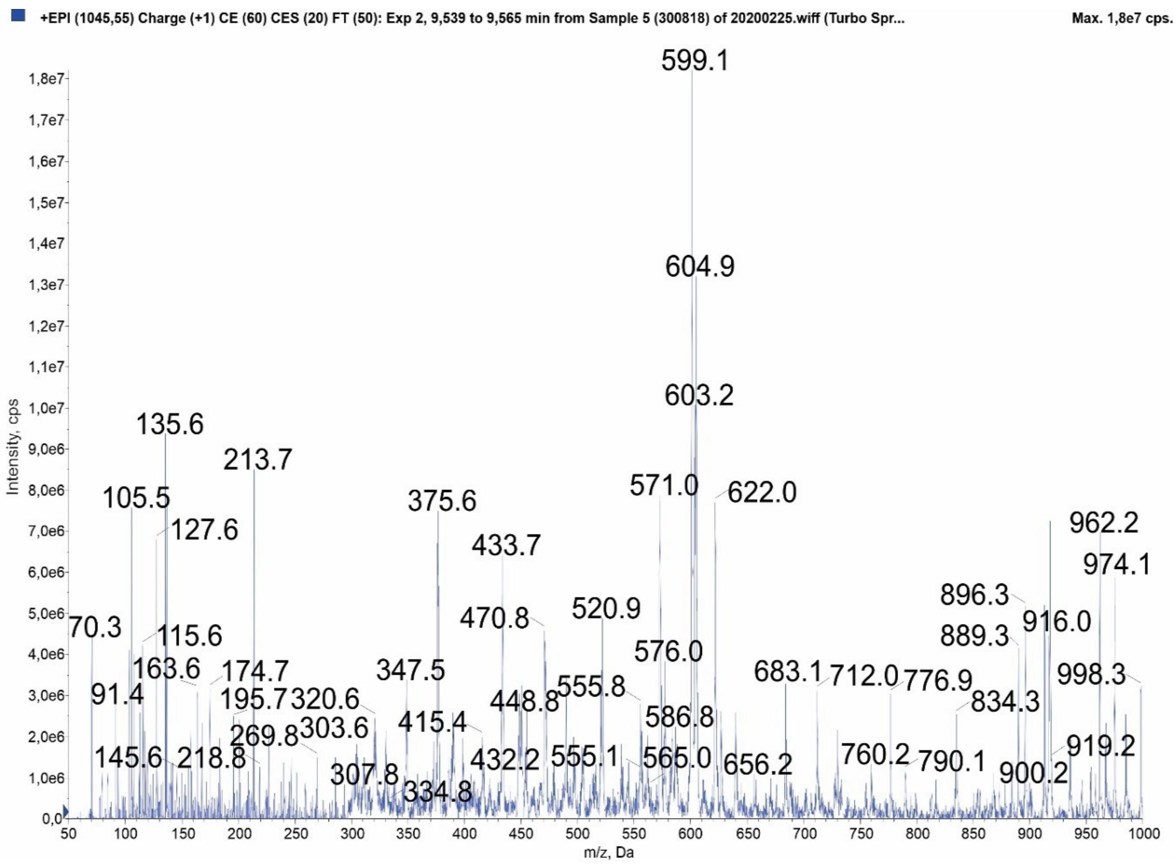


**Figure S8.** Enhanced product ion mass spectrum of microcystin with the suggested structure MC-YR (*m*/*z* 1045) and the following fragment ions: *m*/*z* 974 [M + H – Ala]; 962 [M + H – Mdha]; 916 [M + H – Glu/Masp]; 889 [M + H – Arg]; 834 [M + H – (Glu + Mdha)]; 760 [M + H – (Masp + Arg)]; 683 [Masp + Arg + Adda + Glu + H – $NH_3$ – CO]; 603 [M + H – (Adda + Glu)]; 599 [Arg + Adda + Glu + H], 576 [Glu + Mdha + Ala + Tyr + Masp + H]; 571 [Arg + Adda + Glu + H – CO]; 520 [Ala + Tyr + Masp + Arg + H]; 470 [Arg + Adda + H]; 448 [Tyr + Masp + Arg + H]; 432 [Tyr + Masp + Arg + H – $NH_3$]; 375 [$C_{11}H_{14}O$ + Glu + Mdha + H]; 347 [Adda fragmen + Glu + Mdha + H – $NH_3$ – CO]; 303 [Masp + Arg – $NH_3$]; 269 [Masp + Arg + H – $NH_3$]; 213 [Glu + Mdha + H]; 195 [Glu + Mdha + H – $H_2O$]; 163 [$C_{11}H_{14}O$ + H]; 135 Adda fragment, 127 [Mdha + Ala + H – CO].

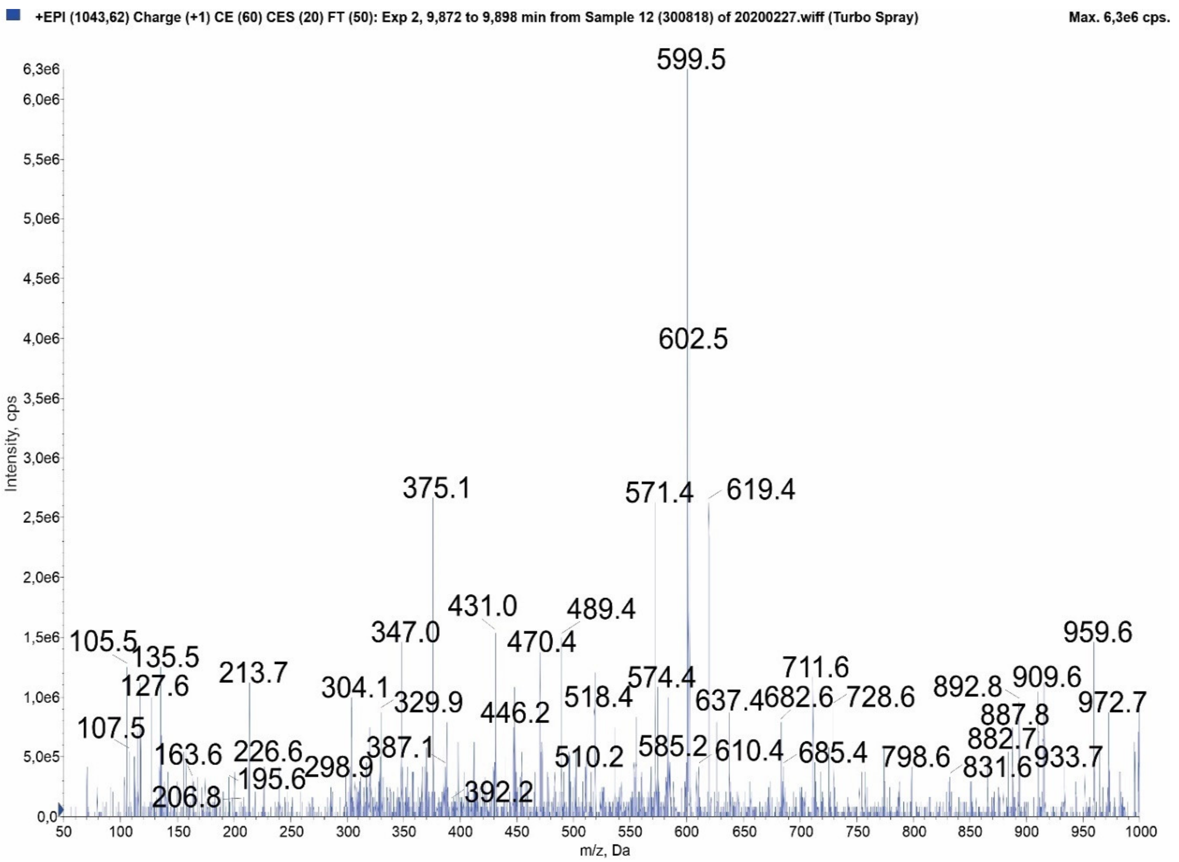


**Figure S9.** Enhanced product ion mass spectrum of microcystin with the suggested structure MC-HphR (*m*/*z* 1043) and the following fragment ions: *m*/*z* 972 [M + H – Ala]; 909 [M + H – Adda fragment]; 892 [$C_{11}H_{14}O$ + Glu + Mdha + Ala + Hph + MeAsp + Arg + H]; 887 [M + H – Arg]; 882 [M + H – Hph]; 831 [M + H – (Glu + Mdha)]; 728 [Masp + Arg + Adda + Glu + H]; 682 [Arg + Adda + Glu + Mdha + H]; 602 [M + H – (Adda + Glu)]; 599 [Arg + Adda + Glu + H]; 574 [M + H – (Adda + Arg)]; 571 [Arg + Adda + Glu + H – CO]; 518 [Ala + Hph + Masp + Arg + H]; 470 [Arg + Adda + H]; 446 [$C_{11}H_{14}O$ + Glu + Mdha + Ala + H]; 375 [$C_{11}H_{14}O$ + Glu + Mdha + H]; 347 [$C_{11}H_{14}O$ + Glu + Mdha + H – CO]; 213 [Glu + Mdha + H]; 195 [Glu + Mdha + H – $H_2O$]; 163 [$C_{11}H_{14}O$ + H]; 135 Adda fragment; 127 [Mdha + Ala + H – CO].

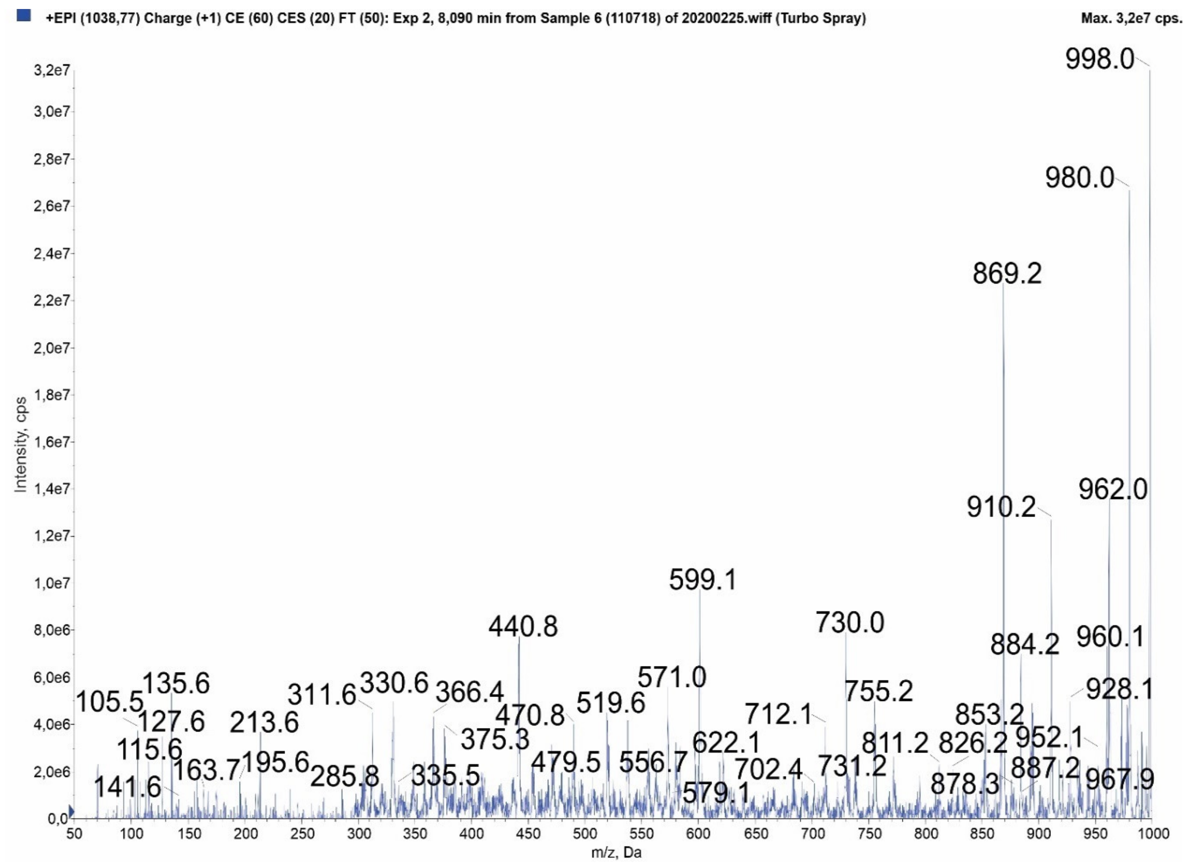


**Figure S10.** Enhanced product ion mass spectrum of microcystin with the suggested structure MC-RR (*m*/*z* 1038/519) and the following fragment ions: *m*/*z* 910 [M + H – Glu/Masp]; 887 [M + H – Arg]; 884 [M + H – (Mdha + Ala)]; 826 [M + H – (Glu + Mdha)]; 811 [M + H – (Ala + Arg)]; 755 [M + H – (Glu + Mdha + Ala)]; 731 [$C_{11}H_{14}O$ + Glu + Mdha + Ala + Arg + Masp + H]; 702 [$C_{11}H_{14}O$ + Glu + Mdha + Ala + Arg + Masp + H – CO]; 599 [Arg + Adda + Glu + H]; 579 [Adda + Glu + Mdha + Ala + H – $H_2O$]; 571 [Arg + Adda + Glu + H – CO]; 470 [Arg + Adda + H]; 440 [Glu + Mdha + Ala + Arg + H]; 375 [$C_{11}H_{14}O$ + Glu + Mdha + H]; 311 [Mdha + Ala + Arg + H]; 285 [Glu + Mdha + Ala + H]; 213 [Glu + Mdha + H]; 195 [Glu + Mdha + H – $H_2O$]; 163 [$C_{11}H_{14}O$ + H]; 135 Adda fragment; 127 [Mdha + Ala + H – CO].

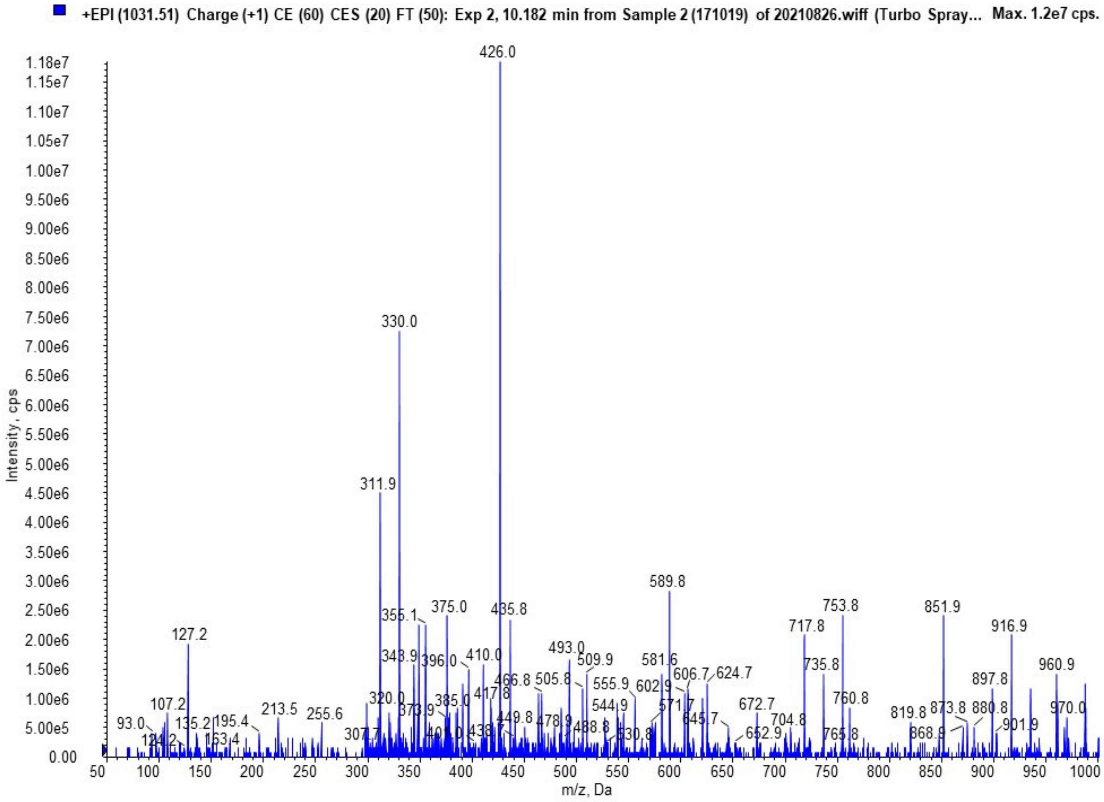


**Figure S11.** Enhanced product ion mass spectrum of microcystin with the suggested structure [Asp$^3$]MC-RY (*m/z* 1031/516) and the following fragment ions: *m/z* 960 [M + H – Ala]; 916 [M + H – Asp]; 897 [M + H – Adda fragment]; 880 [$C_{11}H_{14}O$ + Glu + Mdha + Ala + Arg + Asp + Tyr + H]; 868 [M + H – Tyr]; 819 [M + H – (Glu + Mdha)]; 760 [M + H – (Arg + Asp)]; 753 [M + H – (Tyr + Asp)]; 735 [M + H – (Tyr + Asp) – $H_2O$]; 717 [$C_{11}H_{14}O$ + Glu + Mdha + Ala + Arg + Asp + H]; 606 [Tyr + Adda + Glu + H]; 602 [$C_{11}H_{14}O$ + Glu + Mdha + Ala + Arg + H]; 589 [Mdha + Ala + Arg + Asp + Tyr + H]; 555 [M + H – (Adda + Glu)]; 435 [Arg + Asp + Tyr + H]; 426 [Mdha + Ala + Arg + Asp + H]; 375 [$C_{11}H_{14}O$ + Glu + Mdha + H]; 343 [Ala + Arg + Asp + H]; 311 [Mdha + Ala + Arg + H]; 213 [Glu + Mdha + H]; 163 [$C_{11}H_{14}O$ + H]; 135 Adda fragment.

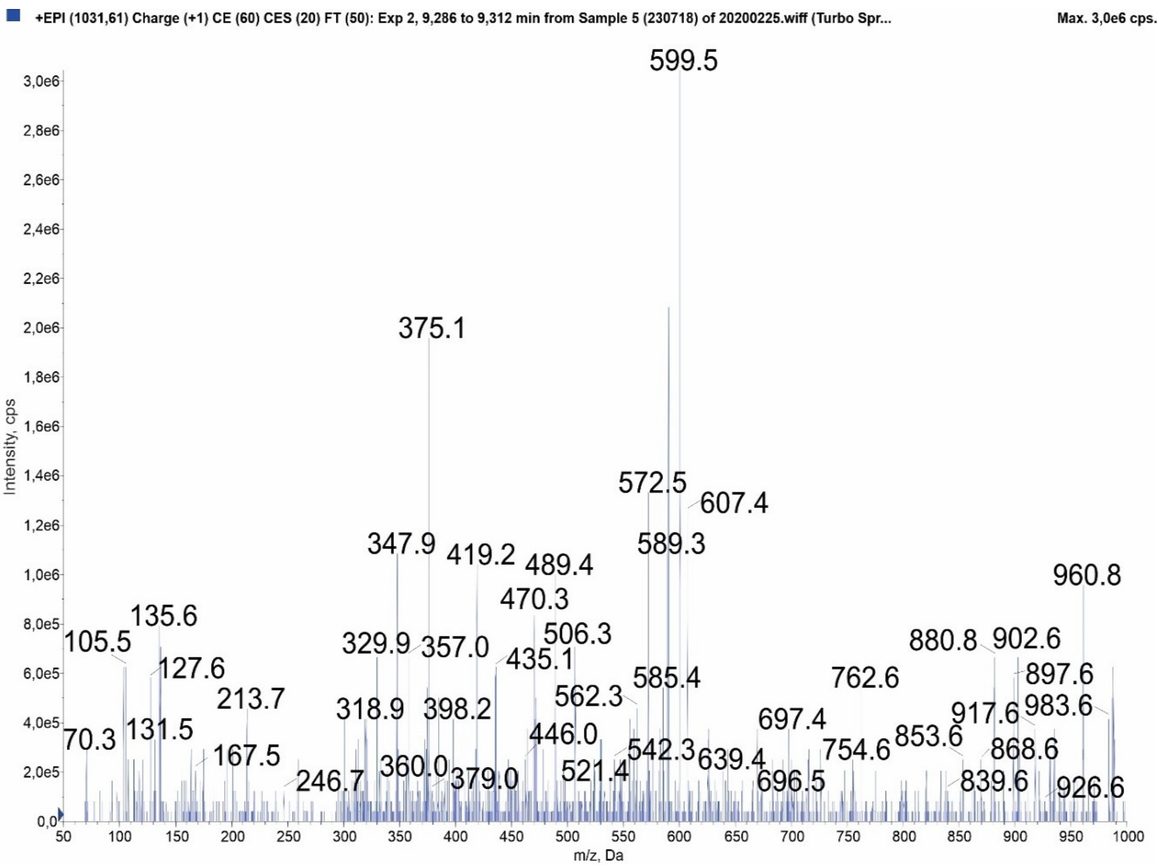


**Figure S12.** Enhanced product ion mass spectrum of microcystin with the suggested structure [Asp$^3$]MC-YR or [Asp$^3$]MC-M($O_2$)R (*m*/*z* 1031/516) and the following fragment ions *m*/*z* 960 [M + H – Ala]; 902 [M + H – Glu/Masp]; 897 [M + H – Adda fragment]; 880 [$C_{11}H_{14}O$ + Glu + Mdha + Ala + Tyr/M($O_2$) + Asp + Arg + H]; 868 [M + H – Tyr/M($O_2$)]; 599 [Arg + Adda + Glu + H]; 589 [Mdha + Ala + Tyr/M($O_2$) + Asp + Arg + H]; 585 [Asp + Arg + Adda + H]; 562 [Glu + Mdha + Ala + Tyr/M($O_2$) + Asp + H]; 506 [Ala + Tyr/M($O_2$) + Asp + Arg + H]; 470 [Arg + Adda + H]; 446 [$C_{11}H_{14}O$ + Glu + Mdha + Ala + H]; 435 [Tyr/M($O_2$) + Asp + Arg + H]; 375 [$C_{11}H_{14}O$ + Glu + Mdha + H]; 357[$C_{11}H_{14}O$ + Glu + Mdha + H – $H_2O$]; 347 [$C_{11}H_{14}O$ + Glu + Mdha + H – CO]; 318 [Mdha + Ala + Tyr/M($O_2$) + H]; 213 [Glu + MeDha + H]; 135 Adda fragment; 127 [Mdha + Ala + H – CO].

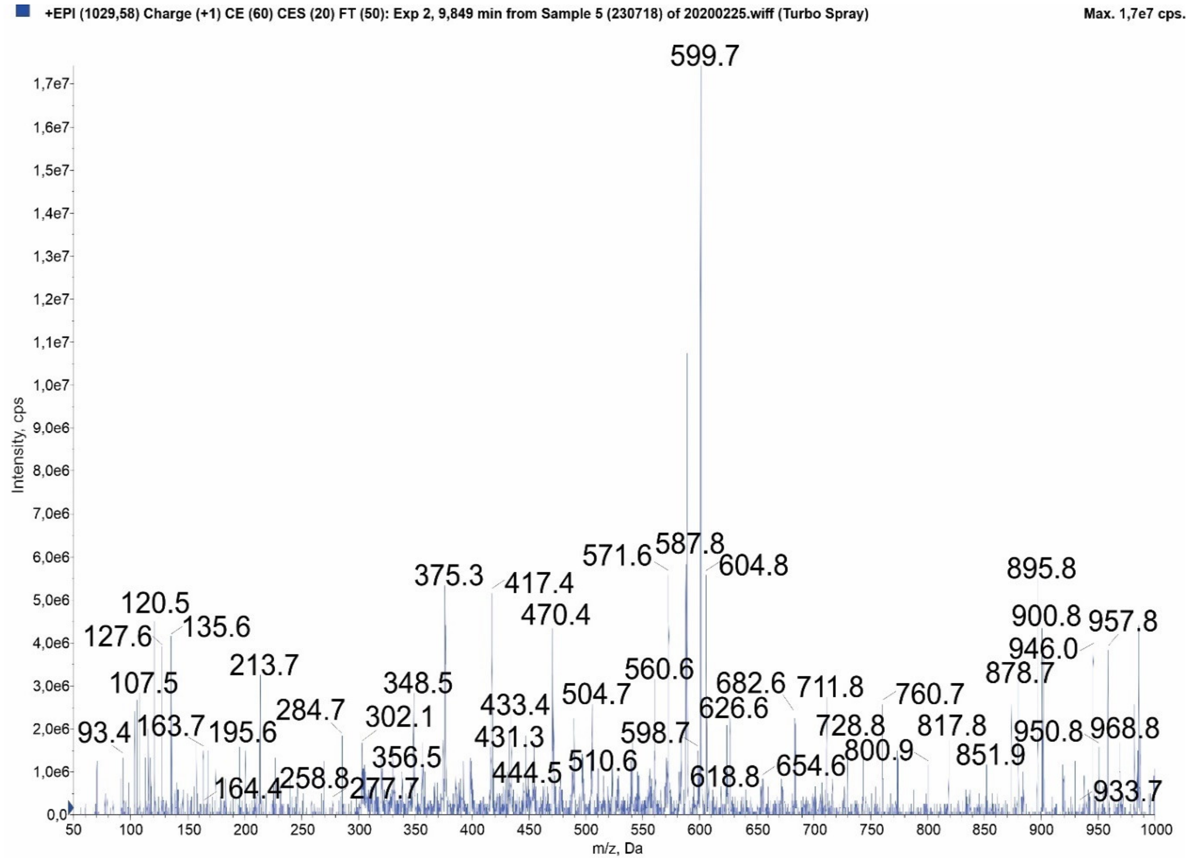


**Figure S13.** Enhanced product ion mass spectrum of microcystin with the suggested structure MC-FR (*m*/*z* 1029) and the following fragment ions *m*/*z* 957 [M + H – Ala]; 946 [M + H – Mdha]; 900 [M + H – Glu/Masp]; 895 [M + H – Adda fragment]; 878 [M + H – Glu/Masp – $H_2O$]; 817 [M + H – (Glu + Mdha)]; 728 [Masp + Arg + Adda + Glu + H]; 682 [Arg + Adda + Glu + Mdha + H]; 604 [M + H – (Adda + Glu) + $NH_3$]; 599 [Arg + Adda + Glu + H]; 587 [M + H – (Adda + Glu)]; 571 [Arg + Adda + Glu + H – CO]; 560 [M + H – (Arg + Adda)]; 504 [Ala + Phe + Masp + Arg + H]; 470 [Arg + Adda + H]; 433 [Phe + Masp + Arg + H]; 431 [Mdha + Ala + Phe + Masp + H]; 375 [$C_{11}H_{14}O$ + Glu + Mdha + H]; 356 [Ala + Phe + Masp + H]; 348 [Ala + Phe + Masp + H]; 302 [Mdha + Ala + Phe + H]; 284 [Glu + Mdha + Ala + H]; 213 [Glu + Mdha + H]; 195 [Glu + Mdha + H – $H_2O$]; 163 [$C_{11}H_{14}O$ + H]; 135 Adda fragment; 127 [Mdha + Ala + H – CO]; 120 Phe immonium ion.

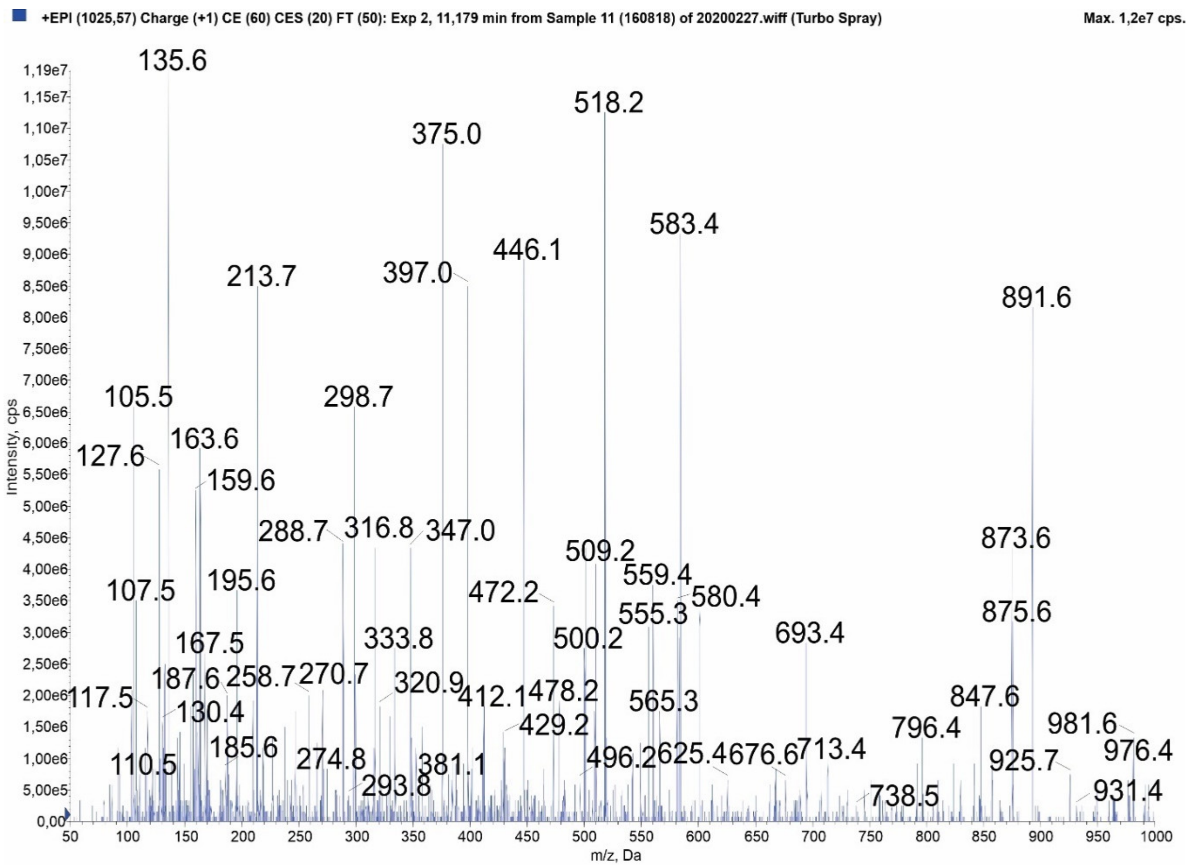


**Figure S14.** Enhanced product ion mass spectrum of microcystin with the suggested structure MC-LW (*m*/*z* 1025) and the following fragment ions: *m*/*z* 976 [M + H – $CH_3OH$ – $NH_3$]; 925 [M + H – Mdha – $NH_3$]; 891 [M + H – Adda fragment]; 873 [$C_{11}H_{14}O$ + Glu + Mdha + Ala + Leu + Masp + Trp + H]; 693 [Adda + Glu + Mdha + Ala + Leu + H – $NH_3$]; 583 [M + H – (Adda + Glu)]; 580 [Adda + Glu + Mdha + Ala + H – $NH_3$]; 565 [Mdha + Ala + Leu + Masp + Trp + H – $H_2O$]; 559 [$C_{11}H_{14}O$ + Glu + Mdha + Ala + Leu + H]; 555 [Mdha + Ala + Leu + Masp + Trp + H – CO]; 509 [Adda + Glu + Mdha + H – $NH_3$]; 500 [Ala + Leu + Masp + Trp + H]; 472 [Ala + Leu + Masp + Trp + H – CO]; 446 [$C_{11}H_{14}O$ + Glu + Mdha + Ala + H]; 429 [Leu + Masp + Trp + H]; 397 [Mdha + Ala + Leu + Masp + H]; 375 [$C_{11}H_{14}O$ + Glu + Mdha + H]; 347 [$C_{11}H_{14}O$ + Glu + Mdha + H – CO]; 316 [Trp + Masp + H]; 298 [Trp + Masp + H – $H_2O$]; 288 [Trp + Masp + H – CO]; 213 [Glu + Mdha + H]; 195 [Glu + Mdha + H – $H_2O$]; 187 [Trp + H]; 163 [$C_{11}H_{14}O$ + H]; 159 Trp immonium ion; 135 Adda fragment; 127 [Mdha + Ala + H – CO].

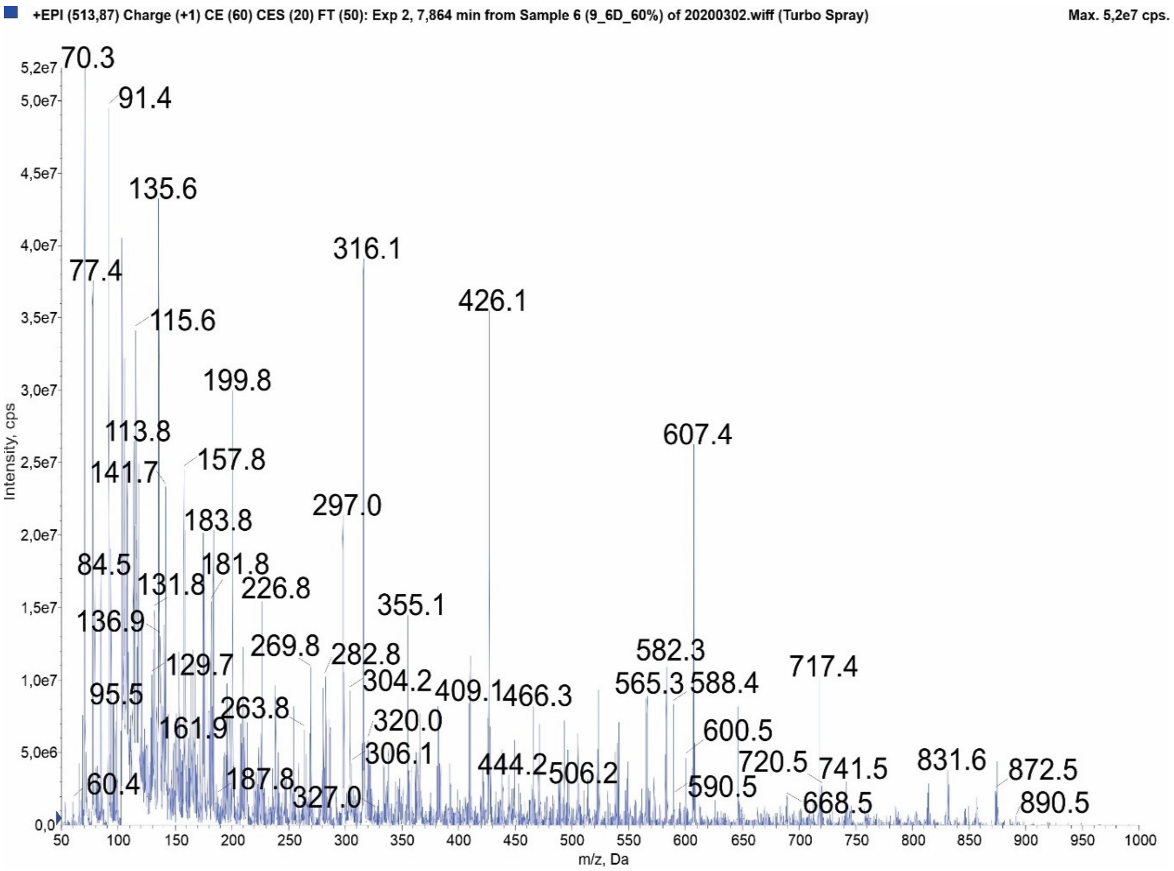


**Figure S15.** Enhanced product ion mass spectrum of microcystin with the suggested structure [Dha$^{7}$]MC-RR (*m*/*z* 1024/512) and the following fragment ions: *m*/*z* 890 [M + H – Adda fragment]; 872 [M + H – Adda fragment – $H_2O$]; 717 [$C_{11}H_{14}O$ + Glu + Dha + Ala + Arg + Masp + H]; 668 [Arg + Adda + Glu + Dha + H]; 588 [$C_{11}H_{14}O$ + Glu + Dha + Ala + Arg + H]; 582 [Dha + Ala + Arg + Masp + Arg + H]; 565 [Dha + Ala + Arg + Masp + Arg + H – $NH_3$]; 426 [Glu + Dha + Ala + Arg + H]; 297 [Dha + Ala + Arg + H]; 269 [Dha + Ala + Arg + H – CO]; 199 [Glu + Dha + H]; 181 [Glu + Dha + H – $H_2O$]; 141 [Dha + Ala + H]; 135 Adda fragment; 113 [Dha + Ala + H – CO].

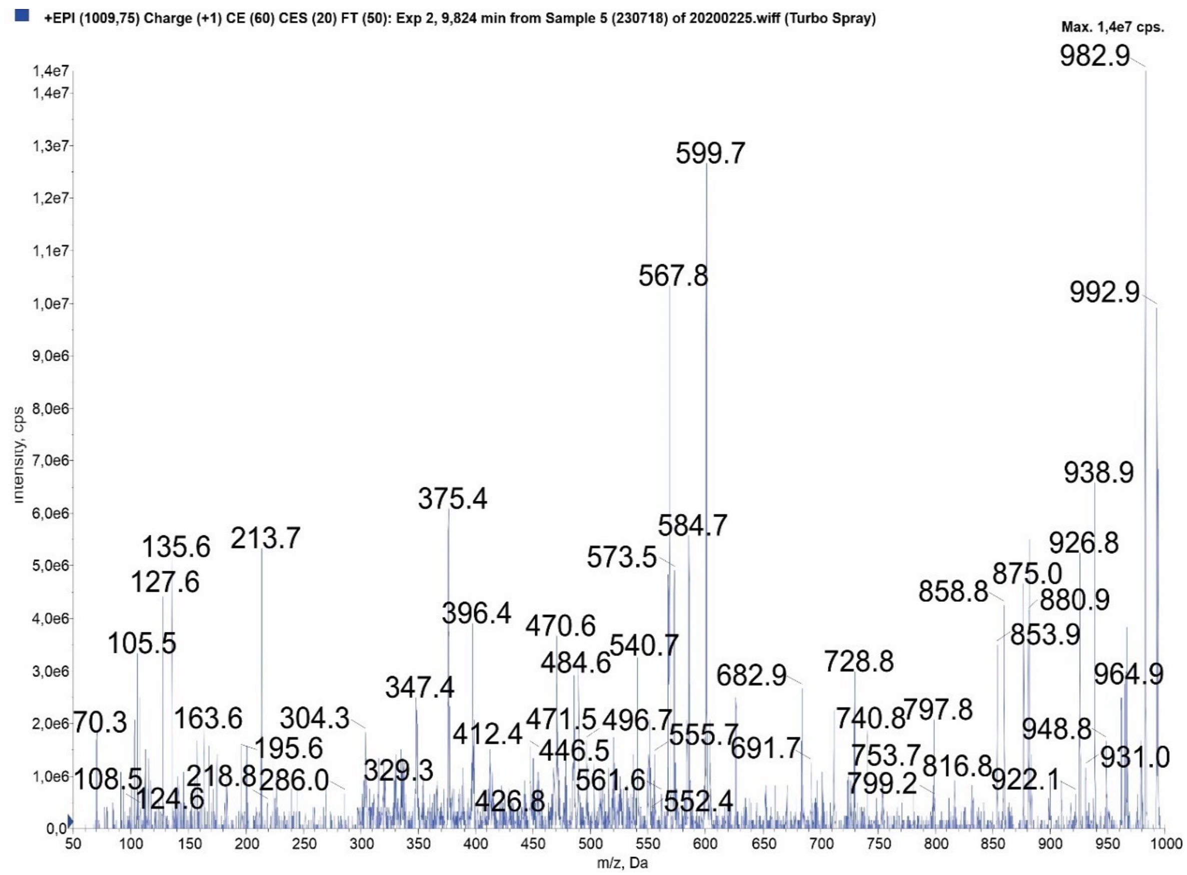


**Figure S16.** Enhanced product ion mass spectrum of microcystin with the suggested structure MC-HilR (*m/z* 1009) and the following fragment ions: *m/z* 992 [M + H – $NH_3$]; 938 [M + H – Ala]; 926 [M + H – Mdha]; 880 [M + H – Glu/Masp]; 875 [M + H – Adda fragment]; 858 [$C_{11}H_{14}O$ + Glu + Mdha + Ala + Hil + Masp + Arg + H]; 853 [M + H – Arg]; 797 [M + H - (Glu + Mdha)]; 753 [M + H – (Hil + Masp); 728 [Masp + Arg + Adda + Glu + H]; 682 [Arg + Adda + Glu + Mdha + H]; 599 [Arg + Adda + Glu + H]; 584 [Mdha + Ala + Hil + Masp + Arg + H + $NH_3$]; 573 [$C_{11}H_{14}O$ + Glu + Mdha + Ala + Hil + H]; 567 [M + H – (Adda + Glu)]; 540 [Glu + Mdha + Ala + Hil + Masp + H]; 484 [Ala + Hil + Masp + Arg + H]; 470 [Arg + Adda + H]; 446 [$C_{11}H_{14}O$ + Glu + Mdha + Ala + H]; 412 [Hil + Masp + Arg + H]; 396 [Hil + Masp + Arg + H – $NH_3$]; 375 [$C_{11}H_{14}O$ + Glu + Mdha + H]; 347 [$C_{11}H_{14}O$ + Glu + Mdha + H – CO]; 213 [Glu + Mdha + H]; 195 [Glu + Mdha + H – $H_2O$]; 163 [$C_{11}H_{14}O$ + H]; 135 Adda fragment; 127 [Mdha + Ala + H – CO].

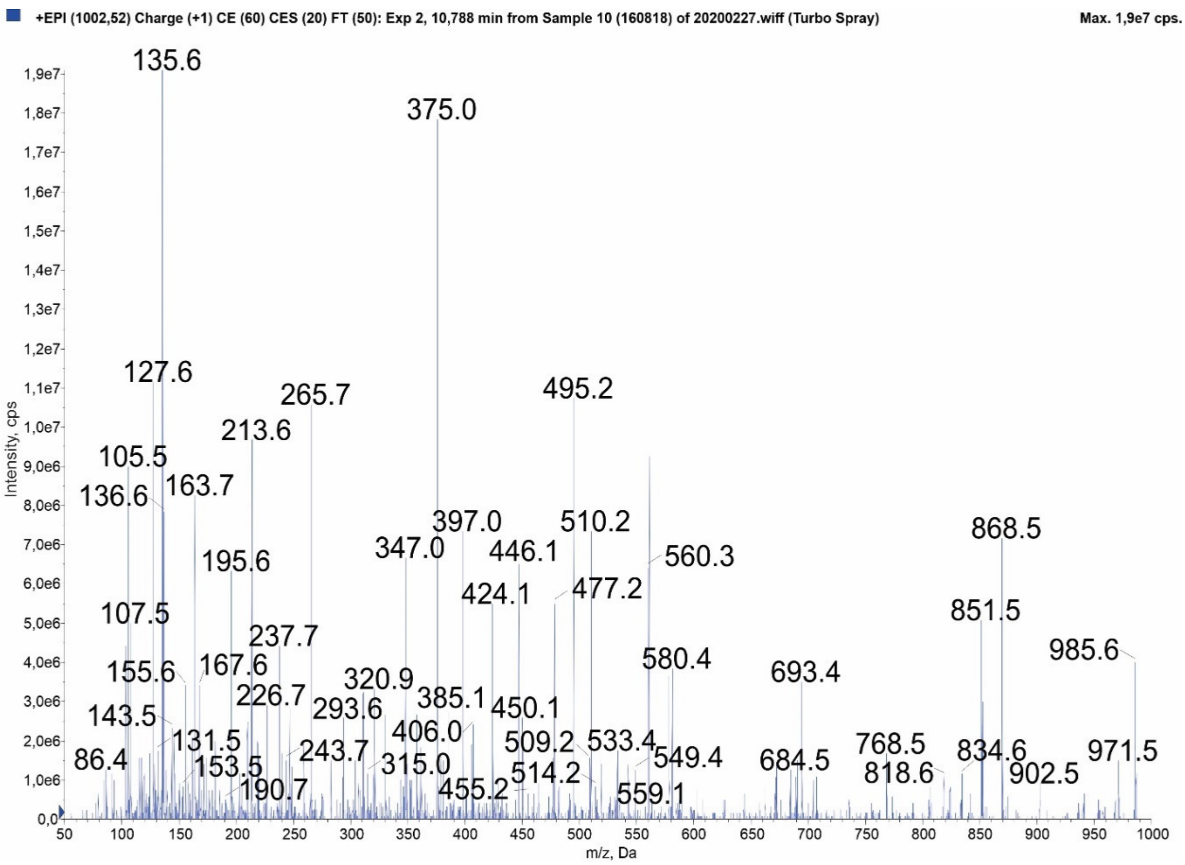


**Figure S17.** Enhanced product ion mass spectrum of microcystin with the suggested structure MC-LY (*m*/*z* 1002) and the following fragment ions: *m*/*z* 985 [M + H – $NH_3$]; 902 [M + H – Mdha – $NH_3$]; 868 [M + H – Adda fragment]; 851 [$C_{11}H_{14}O$ + Glu + Mdha + Ala + Leu + Masp + Tyr + H]; 818 [M + H – (Ala + Leu)]; 693 [Adda + Glu + Mdha + Ala + Leu + H – $NH_3$]; 580 [Adda + Glu + Mdha + Ala + H – $NH_3$]; 560 [M + H – (Adda + Glu)]; 559 [$C_{11}H_{14}O$ + Glu + Mdha + Ala + Leu + H]; 509 [Adda + Glu + Mdha + H – $NH_3$]; 477 [Tyr + Adda + H]; 446 [$C_{11}H_{14}O$ + Glu + Mdha + Ala + H]; 406 [Leu + Masp + Tyr + H]; 397 [Mdha + Ala + Leu + Masp + H]; 375 [$C_{11}H_{14}O$ + Glu + Mdha + H]; 347 [$C_{11}H_{14}O$ + Glu + Mdha + H – CO]; 293 [Masp + Tyr + H]; 213 [Glu + Mdha + H]; 195 [Glu + Mdha + H – $H_2O$]; 163 [$C_{11}H_{14}O$ + H]; 155 [Mdha + Ala + H]; 136 Tyr immonium ion; 135 Adda fragment; 127 [Mdha + Ala + H – CO]; 86 Ile immonium ion.

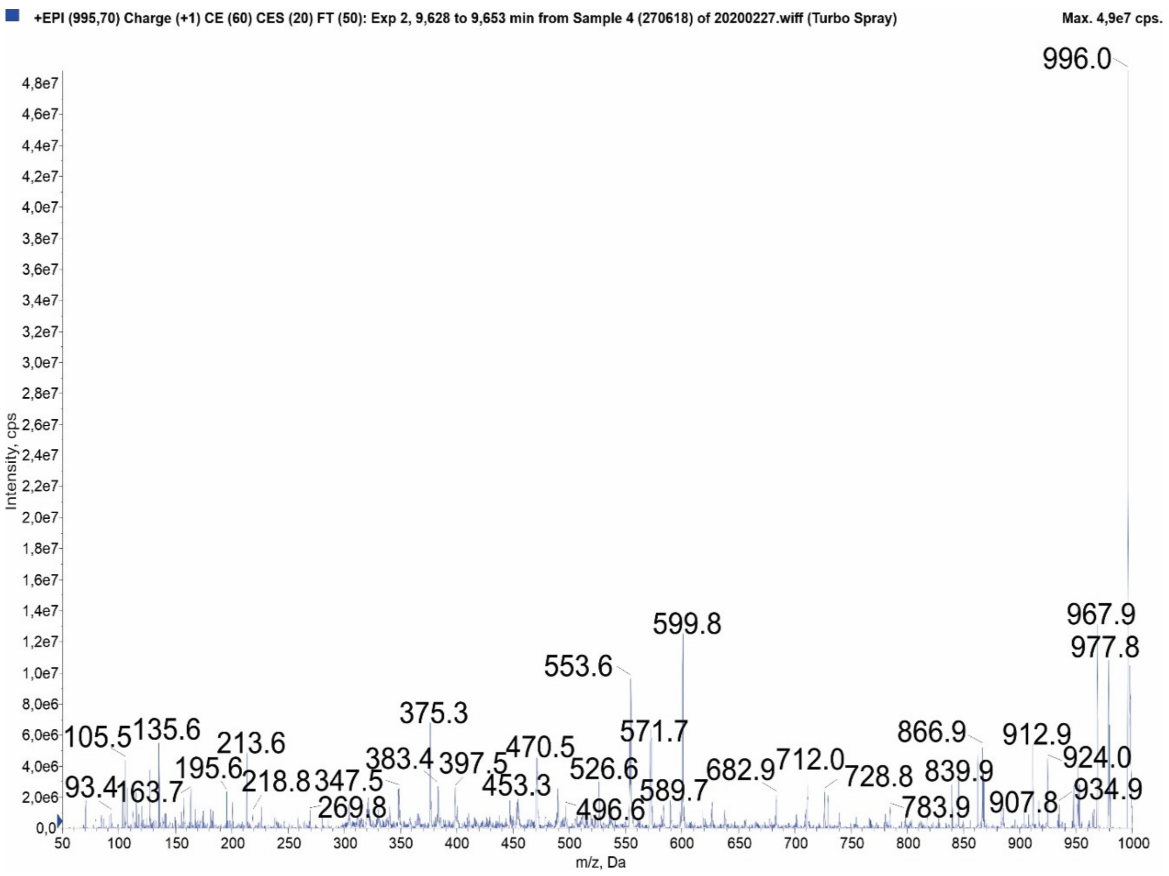


**Figure S18.** Enhanced product ion mass spectrum of microcystin with the suggested structure MC-LR (*m*/*z* 995) and the following fragment ions: *m*/*z* 977 [M + H – $H_2O$]; 967 [M + H – CO]; 924 [M + H – Ala]; 912 [M + H – Mdha]; 866 [M + H – Glu/Masp]; 839 [M + H – Arg]; 783 [M + H – (Glu + Mdha)]; 728 [Masp + Arg + Adda + Glu + H]; 712 [M + H – (Glu + Mdha + Ala)]; 682 [M + H – (Ala + Leu + Masp)]; 599 [Arg + Adda + Glu + H]; 571 [Arg + Adda + Glu + H – CO]; 553 [M + H – (Adda + Glu)]; 526 [Adda + Glu + Mdha + H]; 470 [Arg + Adda + H]; 453 [Ala + Leu + Masp + Arg + H – $NH_3$]; 397 [Glu + Mdha + Ala + Leu + H]; 375 [$C_{11}H_{14}O$ + Glu + Mdha + H]; 347 [$C_{11}H_{14}O$ + Glu + Mdha + H – CO]; 269 [Masp + Arg + H – $NH_3$]; 213 [Glu + Mdha + H]; 195 [Glu + Mdha + H – $H_2O$]; 163 [$C_{11}H_{14}O$ + H]; 135 Adda fragment.

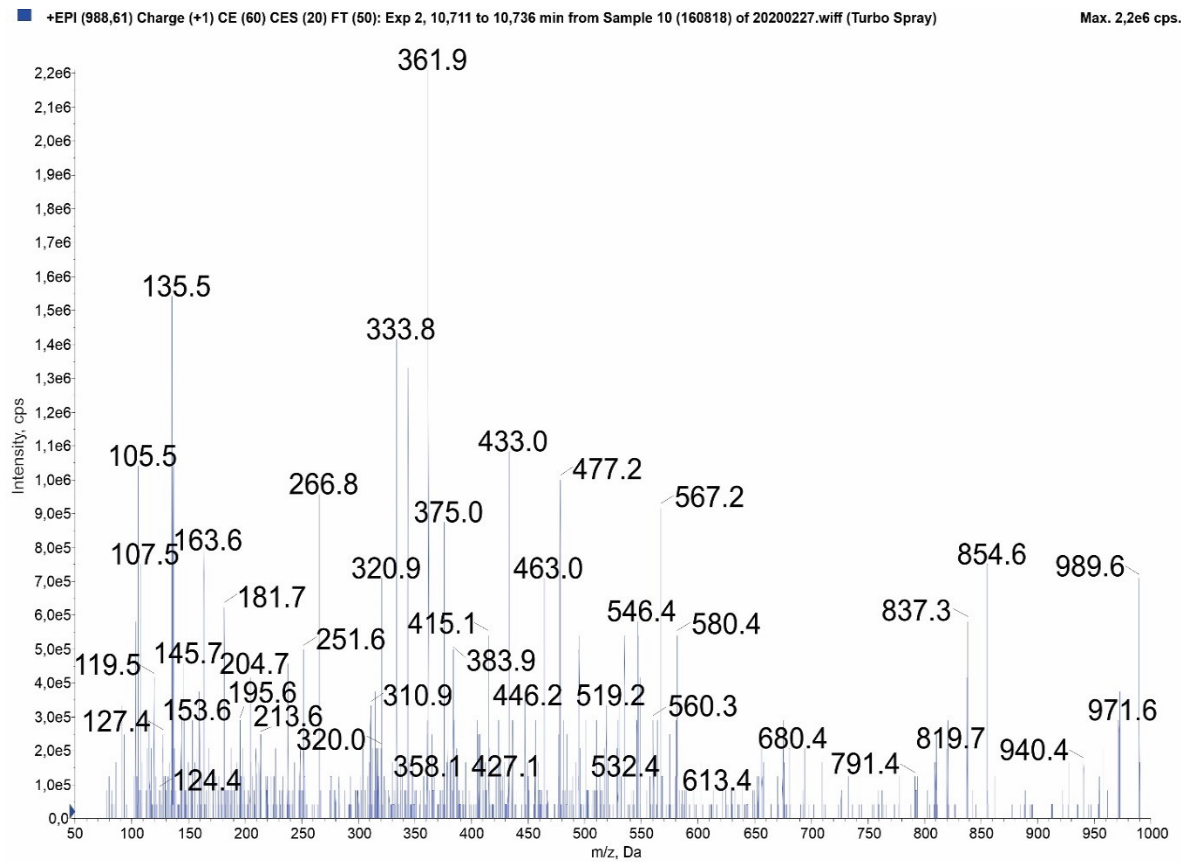


**Figure S19.** Enhanced product ion mass spectrum of microcystin with the suggested structure [Asp$^3$]MC-LY (*m*/*z* 988) and the following fragment ions: *m*/*z* 854 [M + H – Adda fragment]; 837 [$C_{11}H_{14}O$ + Glu + Mdha + Ala + Leu + Asp + Tyr + H]; 580 [Adda + Glu + Mdha + Ala + H – $NH_3$]; 546 [M + H – (Adda + Glu)]; 477 [Tyr + Adda + H]; 463 [Ala + Leu + Asp + Tyr + H]; 446 [$C_{11}H_{14}O$ + Glu + Mdha + Ala + H]; 383 [Mdha + Ala + Leu + Asp + H]; 375 [$C_{11}H_{14}O$ + Glu + Mdha + H]; 266 [Glu + Mdha + Ala + H – $H_2O$]; 251 [Asp + Tyr + H – CO]; 213 [Glu + Mdha + H]; 195 [Glu + Mdha + H – $H_2O$]; 163 [$C_{11}H_{14}O$ + H]; 135 Adda fragment.

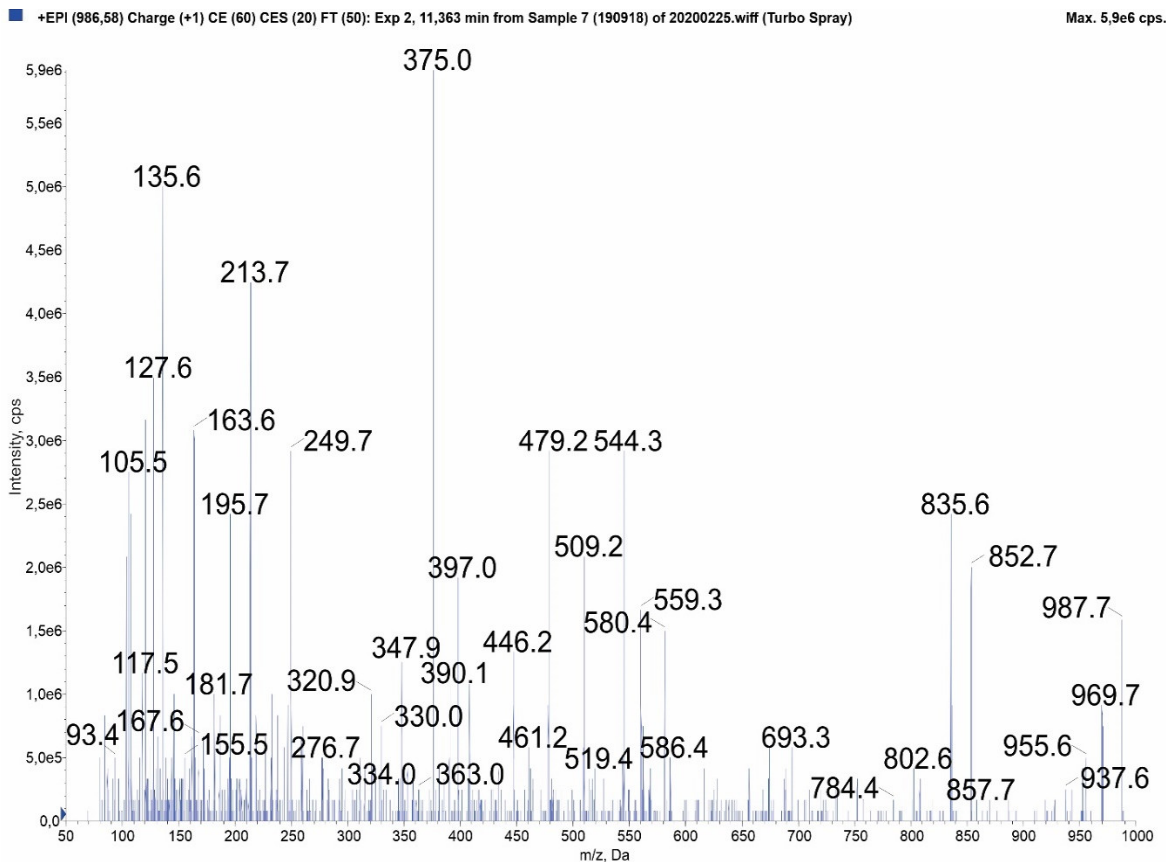


**Figure S20.** Enhanced product ion mass spectrum of microcystin with the suggested structure MC-LF (*m*/*z* 986) and the following fragment ions: *m*/*z* 969 [M + H – $NH_3$]; 857 [M + H – Glu/Masp]; 852 [M + H – Adda fragment]; 802 [M + H – (Ala + Leu)]; 693 [Adda + Glu + Mdha + Ala + Leu + H – $NH_3$]; 580 [Adda + Glu + Mdha + Ala + H – $NH_3$]; 559 [$C_{11}H_{14}O$ + Glu + Mdha + Ala + Leu + H]; 544 [M + H – (Adda + Glu)]; 509 [Glu + Mdha + Ala + Leu + Masp + H – $NH_3$]; 461 [Ala + Leu + Masp + Phe + H]; 446 [$C_{11}H_{14}O$ + Glu + Mdha + Ala + H]; 397 [Glu + Mdha + Ala + Leu + H]; 390 [Leu + Masp + Phe + H]; 375 [$C_{11}H_{14}O$ + Glu + Mdha + H]; 347 [$C_{11}H_{14}O$ + Glu + Mdha + H – CO]; 249 [Masp + Phe + H – CO]; 213 [Glu + Mdha + H]; 195 [Glu + Mdha + H – $H_2O$]; 163 [$C_{11}H_{14}O$ + H]; 135 Adda fragment; 127 [Mdha + Ala + H – CO].

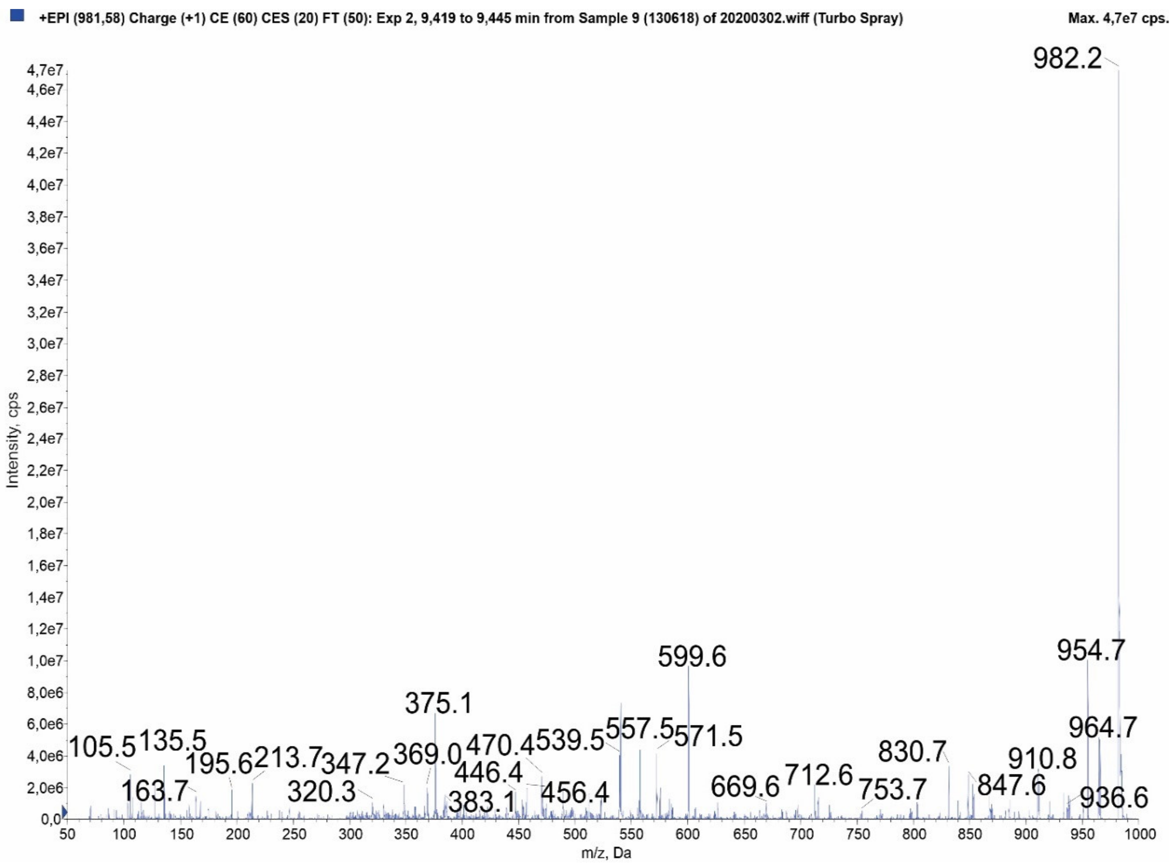


**Figure S21.** Enhanced product ion mass spectrum of microcystin with the suggested structure [Asp$^{3}$]MCLR (*m*/*z* 981) and the following fragment ions: *m*/*z* 910 [M + H – Ala]; 847 [M + H – Adda fragment]; 830 [$C_{11}H_{14}O$ + Glu + Mdha + Ala + Leu + Asp + Arg + H]; 753 [Arg + Adda + Glu + Mdha + H]; 668 [M + H – Adda]; 599 [Arg + Adda + Glu + H]; 571 [Arg + Adda + Glu + H – CO]; 539 [M + H – (Adda + Glu)]; 470 [Arg + Adda + H]; 456 [M + H – (Adda + Glu + Mdha)]; 446 [$C_{11}H_{14}O$ + Glu + Mdha + Ala + H]; 383 [Mdha + Ala + Leu + Asp + H]; 375 [$C_{11}H_{14}O$ + Glu + Mdha + H]; 369 [Glu + Mdha + Ala + Leu + H – CO]; 347 [$C_{11}H_{14}O$ + Glu + Mdha + H – CO]; 213 [Glu + Mdha + H]; 195 [Glu + Mdha + H – $H_2O$]; 163 [$C_{11}H_{14}O$+H]; 135 Adda fragment; 105 Dha.

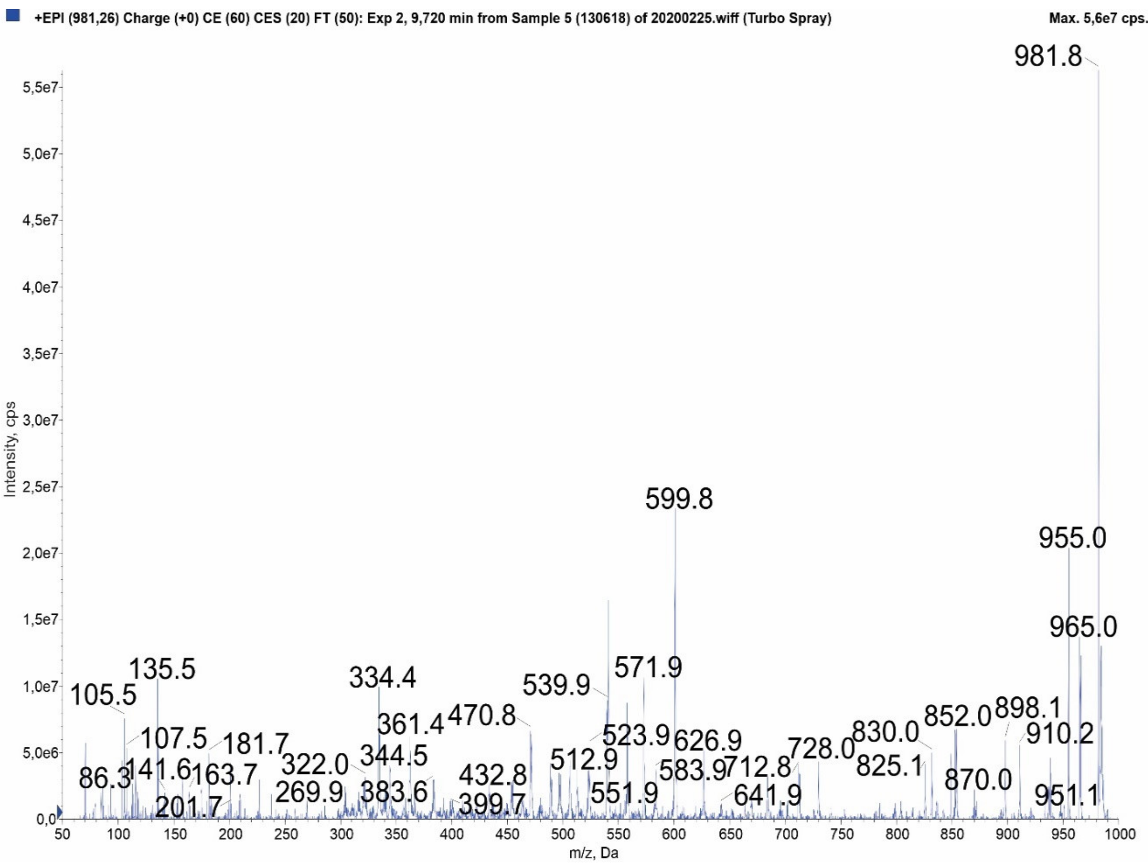


**Figure S22.** Enhanced product ion mass spectrum of microcystin with the suggested structure [Dha$^7$]MCLR (*m*/*z* 981) and the following fragment ions: *m*/*z* 910 [M + H – Ala]; 852 [M + H – Glu/Mdha]; 830 [$C_{11}H_{14}O$ + Glu + Dha + Ala + Leu + Masp + Arg + H]; 825 [M + H – Arg]; 728 [Masp + Arg + Adda + Glu + H]; 712 [Leu + Masp + Arg + Adda + H]; 599 [Arg + Adda + Glu + H]; 583 [Adda + Glu + Dha + Ala + H]; 571 [Arg + Adda + Glu + H – CO]; 539 [M + H – (Adda + Glu)]; 512 [Adda + Glu + Dha + H]; 470 [Arg + Adda + H]; 432 [$C_{11}H_{14}O$ + Glu + Dha + Ala + H]; 399 [Leu + Masp + Arg + H]; 383 [Glu + Dha + Ala + Leu + H]; 361 [$C_{11}H_{14}O$ + Glu + Dha + H]; 334 [$C_{11}H_{14}O$ + Glu + Dha + H – CO]; 269 [Glu + Dha + Ala + H]; 181 [Glu + Dha + H – $H_2O$]; 163 [$C_{11}H_{14}O$ + H]; 141 [Dha + Ala + H]; 135 Adda fragment.
